# The emerging role of CARM1 in cancer

**DOI:** 10.1007/s13402-024-00943-9

**Published:** 2024-04-15

**Authors:** Zizhuo Xie, Yuan Tian, Xiaohan Guo, Na Xie

**Affiliations:** West China School of Basic Medical Sciences and Forensic Medicine, Sichuan University, and Collaborative Innovation Center for Biotherapy, Chengdu, 610041 China

**Keywords:** CARM1, Arginine methylation, Tumorigenesis, Therapeutic target, Biomarker

## Abstract

Coactivator-associated arginine methyltransferase 1 (CARM1), pivotal for catalyzing arginine methylation of histone and non-histone proteins, plays a crucial role in developing various cancers. CARM1 was initially recognized as a transcriptional coregulator by orchestrating chromatin remodeling, transcription regulation, mRNA splicing and stability. This diverse functionality contributes to the recruitment of transcription factors that foster malignancies. Going beyond its established involvement in transcriptional control, CARM1-mediated methylation influences a spectrum of biological processes, including the cell cycle, metabolism, autophagy, redox homeostasis, and inflammation. By manipulating these physiological functions, CARM1 becomes essential in critical processes such as tumorigenesis, metastasis, and therapeutic resistance. Consequently, it emerges as a viable target for therapeutic intervention and a possible biomarker for medication response in specific cancer types. This review provides a comprehensive exploration of the various physiological functions of CARM1 in the context of cancer. Furthermore, we discuss potential CARM1-targeting pharmaceutical interventions for cancer therapy.

## Introduction

**Fig. 1:**
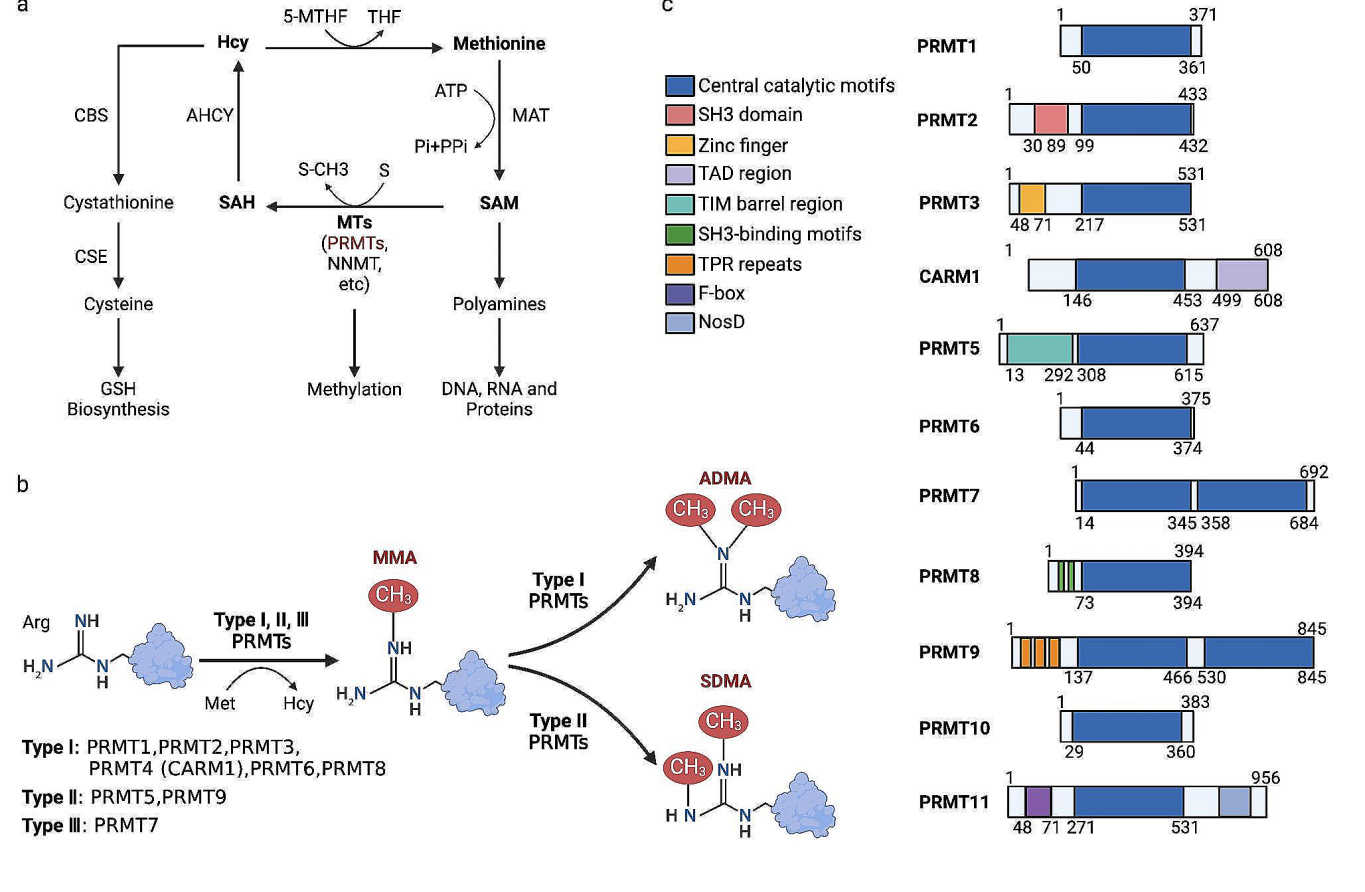
The mammalian PRMTs and related metabolic processes. (**a**) The universal methyl donor S-adenosyl-methionine (SAM) is enzymatically synthesized from methionine through the catalytic action of methionine adenosyltransferase 2A (MAT2A). SAM is consumed by PRMTs, a subclass of methyltransferases (MTs), to effectuate the methylation of arginine, thereby producing S-adenosyl-homocysteine (SAH). SAH, in turn, undergoes recycling back into methionine by methionine synthase (MS) or used in the transsulfuration pathway for glutathione production. (**b**) PRMTs exhibit the remarkable capacity to catalyze various methylation reactions on arginine, encompassing monomethylation (MMA), asymmetric dimethylation (ADMA), and symmetric dimethylation (SDMA), all facilitated by SAM as methyl donor. Type I PRMTs, namely PRMT1-4, PRMT6, and PRMT8, predominantly catalyze MMA and ADMA. On the other hand, type II enzymes, PRMT5 and PRMT9, are responsible for catalyzing both MMA and SDMA. Type III, such as PRMT7, specifically mediates the MMA. (**c**) Eleven distinct PRMTs have been identified, each featuring a typical SAM-dependent MTase catalytic core domain and diverse N-terminal non-catalytic domains. Abbreviations: ADMA, asymmetric dimethylation (ADMA); GSH, reduced glutathione; MAT2A, methionine adenosyltransferase 2A; MMA, monomethylation; MTAP, methylthioadenosine phosphorylase; MTR, methylthioribose; MTs, Methyltransferases, SAH, S-adenosyl-homocysteine (SAH); SAM, S-adenosyl-methionine; SDMA, symmetric demethylation; THF, tetra-hydrofolate

Protein methylation modification is a common post-translational modification (PTM) closely related to cancer incidence and progression. This PTM is precisely controlled by protein methyltransferases (writers) and demethylases (erasers). The enzyme that catalyzes the methylation modification of arginine is called protein arginine methyltransferase (PRMT), which has nine members, namely PRMT1-9. Using S-adenosylmethionine (AdoMet/SAM) as a methyl donor, PRMTs can catalyze monomethylation (Type III isoform: PRMT7), symmetric dimethylation (Type II isoforms: PRMT5, PRMT5), and asymmetric dimethylation (Type I isoforms: PRMT1-4, PRMT6, PRMT8) (Fig. 1) [1]. By changing the hydrophobicity, increasing the size of the side chain, and reducing the hydrogen bond potential, arginine methylation generates a docking site for effector proteins (reader), which include plant homeodomain (PHD) zinc finger, Tudor domain and SH3 domain [2]. Therefore, protein methylation affects the stability, localization, activity, and interaction of substrate proteins, which participate in a variety of physiological and pathological processes [3, 4].

PRMT4 also known as coactivator-associated arginine methyltransferase 1 (CARM1), belongs to the type I PRMTs. This enzyme exhibits the capability to monomethylate and asymmetrically methylate the guanosine-nitrosyl group of arginine residues in target proteins [5]. CARM1 is known for its distinctive substrate specificity, as it typically methylates substrates within a loosely defined proline/glycine/methionine motif, distinguishing itself from other PRMTs that target glycine/arginine-rich motifs [6]. The non-overlapping functions of CARM1 and other PRMTs render CARM1 an appealing therapeutic target for various diseases. Several recent reviews have explored CARM1’s potential as a therapeutic target in cancers, shedding light on its role in the pathogenesis of various malignancies [3, 5]. In this review, our objective is to highlight the impact of CARM1’s functional diversity in carcinogenesis, particularly how it may be influenced by the variable expression of its alternatively spliced isoforms. Furthermore, we delve into the regulatory mechanisms exerted by oncogenic signals on the expression, PTMs and enzymatic activity of CARM1 across different cancer types. Our exploration extends beyond CARM1’s initial characterization as a transcriptional coactivator, revealing its critical involvement in diverse physiological processes, such as oxidative stress, cell death, and metabolism, contributing to tumor development, metastasis, and therapeutic resistance. This comprehensive understanding of CARM1’s multifaceted functions positions it as a promising target for therapeutic interventions in the complex landscape of cancer biology.

## Dysregulated arginine methylation by CARM1 in tumor

**Fig. 2:**
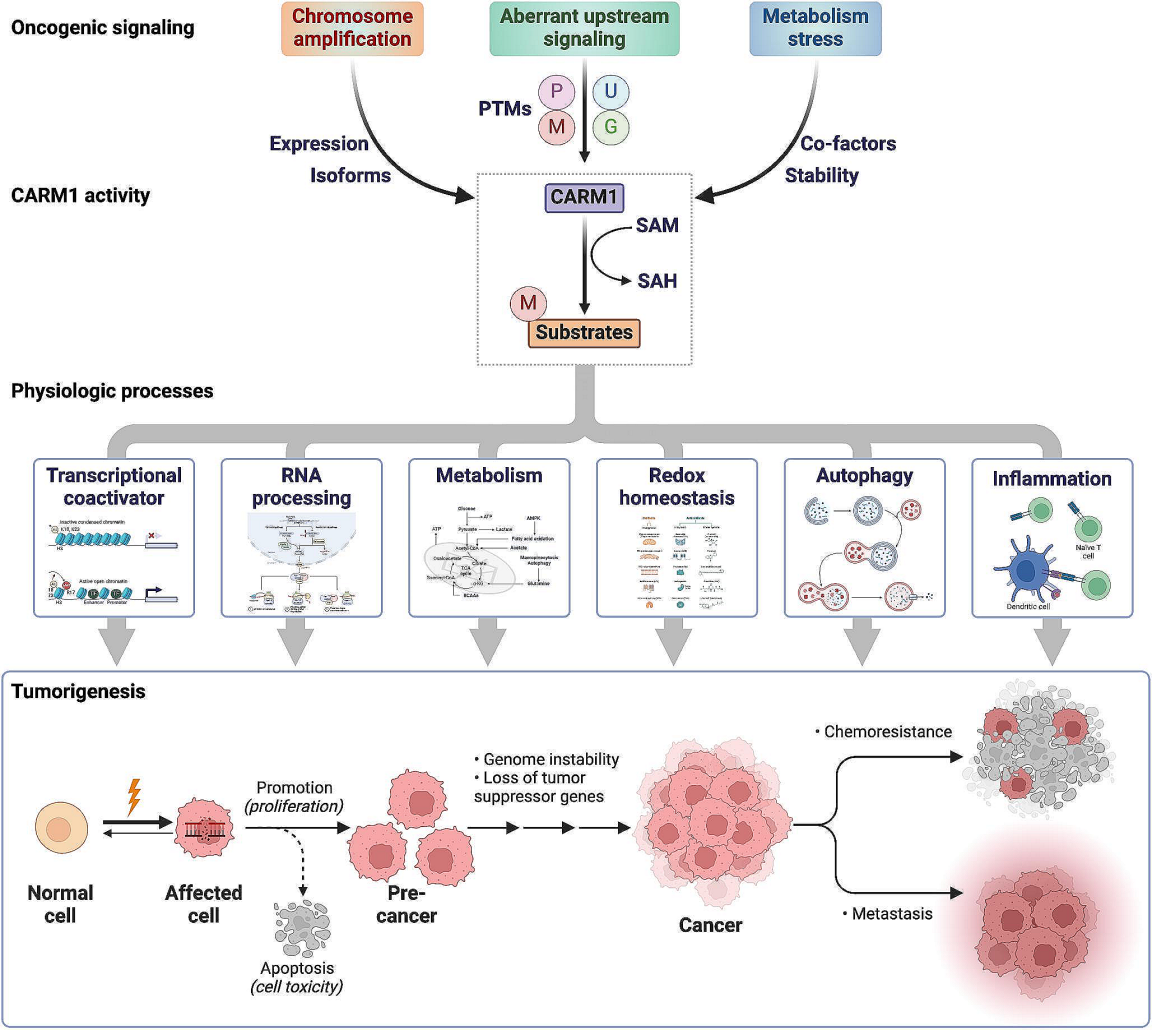
The overview of CARM1-mediated methylation in oncogenic processes. CARM1 is a sensor for oncogenic signals, nutrients, and oxidative stress, which control tumorigenesis, resistance and metastasis. The overexpressed or overactivated CARM1 catalyzes the methylation of target proteins, which implicates in various pathways, including transcriptional activation, RNA processing, metabolism, redox homeostasis, autophagy and inflammation

Oncogenic CARM1 exhibits varying expression levels across different cancer types. Through selective splicing, CARM1 manifests in several isoforms, including the full-length variant (referred to as CARM1) that is predominantly expressed in healthy heart, brain, testis, and skeletal muscle, and the truncated CARM1 lacking exon 15 (CARM1ΔE15) that prevails in breast cancers. The distinct activity profiles of these two isoforms, characterized by differential subcellular localization, substrate methylation levels, and binding affinities with target proteins, contribute to the diverse enzymatic activities of CARM1 in various tissues. Moreover, the enzymatic activity of CARM1 undergoes significant changes depending on its isoforms and PTMs within the diverse cancer landscape (Fig. 2). Oncogenic signals exert control over the PTMs of CARM1, including phosphorylation, methylation, ubiquitination, and O-GlcNAcylation. These modifications alter its homodimerization, SAM binding activity, substrate specificity, subcellular localization, and stability. Additionally, the altered expression of demethylases, like lysine-specific demethylase 1 (LSD1) and Jmjc-domain-containing protein 6 (JMJD6), also contributes to the dysregulated arginine methylation by CARM1 in tumorigenesis.

### Alternatively expression pattern of CARM1 isoforms

**Fig. 3:**
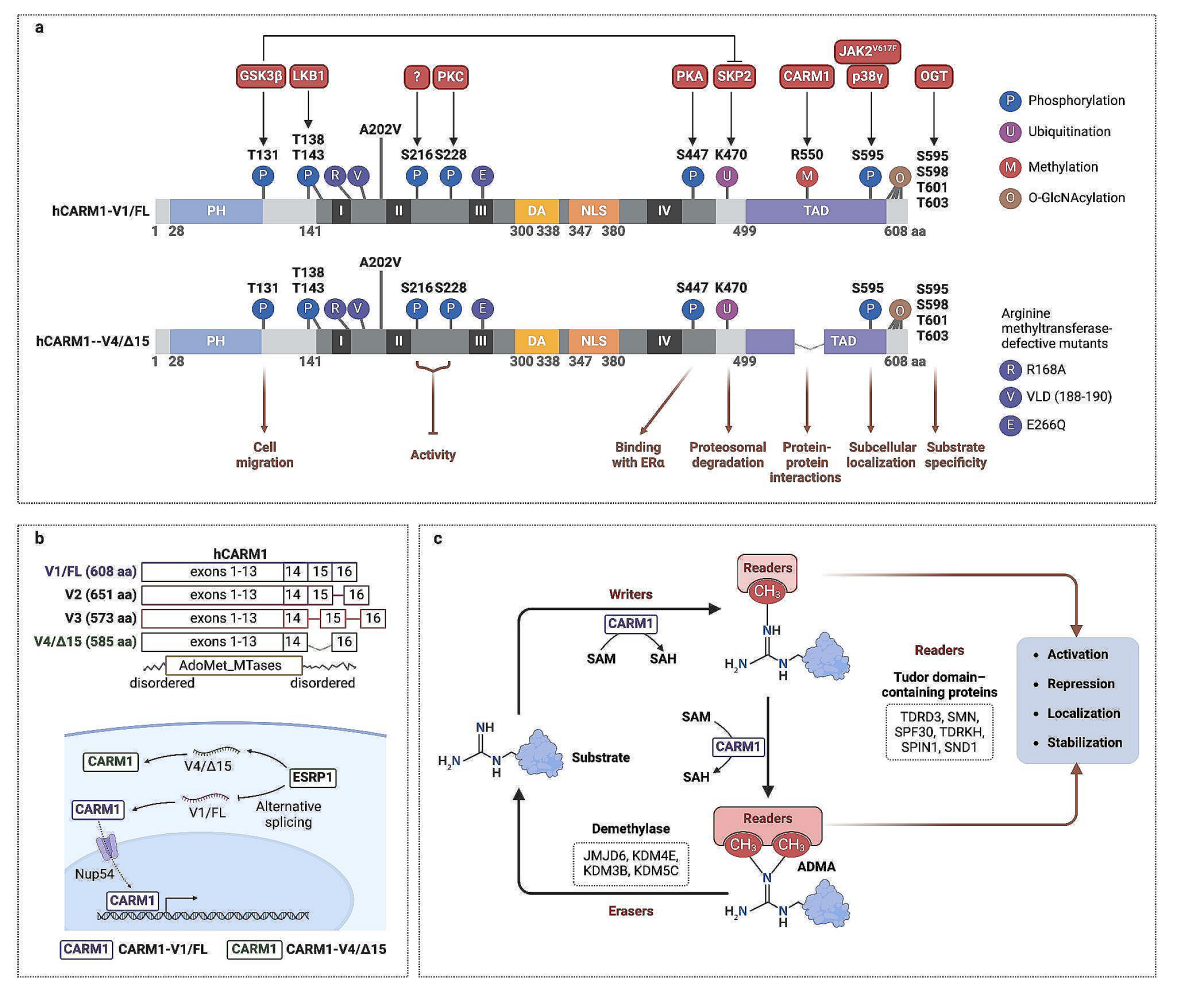
Regulating the activity and function of CARM1 by post-translational modifications. (**a**) The full-length CARM1 with 608 amino acids has been schematically divided into three domains essential for its function: including the N-terminal domain of CARM1 contains a pleckstrin homology (PH)-like domain with the arginine methyltransferase activity, the C-terminal domain of CARM1 (residues 479–608 in mCARM1) contains multiple motifs involved in protein-protein interactions, and the flexible linker region that contains a catalytic core domain (residues 149–469). Multiple post-translational modifications, like phosphorylation, ubiquitination, methylation, and O-GlcNAcylation, of CARM1 can alter its activity and function in response to various physiological and environmental stimuli. (**b**) CARM1 has several alternatively spliced isoforms, including the full-length isoform (V1/FL) with 608 aa, V2 isoform with 651 aa, V3 isoform with 573 aa and V4/ΔE15 isoform with 585 aa, respectively. ESRP1 regulates the alternative splicing of CARM1, resulting in reduced CARM1 and increased CARM1ΔE15. The CARM1 protein can be imported by Nup54 into nuclear, where it serves as the transcriptional activator. (**c**) CARM1 serves as an arginine methyltransferase enzyme writer that adds mono- and asymmetric dimethylation to the arginyl residues in target proteins using SAM as the methyl donor. Demethylases can reversibly demethylate this modification, termed the ‘erasers’. Methylarginines, which regulate the pleiotropic biological functions, are further recognized by ‘readers.’ *Abbreviations:* ADMA, asymmetric dimethylation; DA, dimerization arm; ESRP1, Epithelial Splicing Regulatory Protein 1; JMJDs, Jumonji C domain-containing proteins; NLS, nuclear localization sequence; Nup54, nucleoporin 54; OGT, O-linked N-acetylglucosamine transferase; PH, pleckstrin homology; TAD, transcriptional activation domain

CARM1 presents diverse isoforms through selective splicing, characterized by differential subcellular localization, stability, substrate methylation levels, and binding affinities with target proteins, contributing to the diverse enzymatic activities of CARM1 in various tissues (Fig. 3). The most frequently reported CARM1 isoform in human tumors is a full-length isoform with 608 amino acids (aa) (referred to as CARM1-V1/FL). The N-terminal domain of full-length CARM1 has arginine methyltransferase activity responsible for adding a methyl group to arginine residues in target proteins. This domain also contains the coactivator protein’s binding site, which helps bring CARM1 to the promoter regions of target genes. The C-terminal domain of CARM1 contains multiple motifs involved in protein-protein interactions, which recruit other transcriptional regulatory proteins to the target gene and help anchor CARM1 to the chromatin. The flexible linker region that contains a highly conserved catalytic core domain (residues 149–469) is believed to facilitate the interaction between CARM1 and its target proteins and regulate CARM1 activity (Fig. 3a) [7].

Compared to the full-length variant, the truncated CARM1 lacking exon 15 (CARM1-V4/ΔE15) in specific cells exhibits different localization and activity, particularly in breast cancers. CARM1ΔE15, being more resistant to degradation, shows a stronger association with nuclear bodies than CARM1. Automethylation of CARM1 is detected only in CARM1 and not in CARM1ΔE15, indicating that exon 15 contains a necessary sequence for sustaining R551 automethylation [8]. Apart from maintaining R551 automethylation, exon 15 contains a crucial region facilitating the interaction of CARM1 with specific target proteins. Consequently, CARM1ΔE15 methylates fewer substrates than CARM1, demonstrating a preferential substrate specificity for transcriptional coactivators. A notable example of alternative splicing-mediated cell type-specific expression of CARM1 isoforms is the preferential expression of CARM1 in the luminal (epithelial) region of normal mouse mammary glands with higher ERα protein levels, while the preferential expression of CARM1ΔE15 is observed in the stroma. Due to the inability of CARM1ΔE15 to activate ERα transcriptional activity, the unique preferential expression of CARM1ΔE15 in the stroma indicates differing roles for ERα in the epithelium and stroma [8]. Furthermore, the distribution of CARM1 and CARM1-ΔE15 proteins differs in breast cancer cells. Specifically, HER2 tumors exhibit a higher concentration of CARM1 protein in the nucleus, while triple-negative breast cancer (TNBC) and HER2 tumors are associated with cytoplasmic expression of CARM1ΔE15 proteins [9, 10]. The alternative splicing of CARM1 can be regulated by epithelial splicing regulatory protein 1 (ESRP1) (Fig. 3b). ESRP1 reduced the ratio of CARM1FL to CARM1ΔE15, rendering small cell lung cancer cells sensitized to chemotherapy [11].

Decides the full-length and ΔE15 variants, another two isoforms have also been identified in rat normal tissues. Compared to the CARM1ΔE15 by skips of exon 15, CARM1-v2 was created by keeping intron 15, leading to the production of deduced CARM1 with 651 aa. The production of CARM1-v3 involved the retention of two intron sequences from the original transcript (1,709-1,986 and 2,056 − 2,184). This led to the insertion of v3-specific amino acid sequences (residues 540–573) in place of the C-terminal amino acids from CARM1-v1 [12]. CARM1-v3, but not the other isoforms, strongly regulates the alternative splice of mRNA. The v3-specific sequences determine the selection of the 5′ alternative splice site, thereby promoting a change in the pre-mRNA E1A minigene’s distal 5’ splice location and improving the exon skipping in the CD44 reporter [12]. Moreover, the various CARM1 transcripts have distinct expression patterns in different tissues. CARM1-v1 is predominantly expressed in healthy hearts, brains, testis, and skeletal muscles, CARM1-v2 can be detected in the liver, brain, and testis. CARM1-v3 is expressed at high levels in the adult kidney, liver, spleen, and fetal brain. The expression levels of CARM1-v4 in the spleen, lung and kidney are comparable to CARM1-v1 (Fig. 3b) [12]. Overall, the diverse expression levels and subcellular localization patterns of various CARM1 isoforms may potentially account for observed discrepancies in the functional roles of CARM1 in cancers.

### PTMs-altered enzymatic activity of CARM1

Oncogenic signals in various cancers intricately modulate the PTMs of CARM1, thereby changing its enzymatic activity and substrate interaction affinities. The PTMs of CARM1, including phosphorylation, methylation, ubiquitination, and O-GlcNAcylation, have been demonstrated to finely tune its enzymatic activity by orchestrating changes in homodimerization, SAM binding activity, substrate specificity and subcellular localization (Table 1).

**Table 1:** Various PTMs determine the enzymatic activity of CARM1

Human AA	Mouse aa	Type of PTMs	Enzymes catalyzing the modification	Effect of PTMs	Refs
S228	S229	Phosphorylation	PKC	Prevents CARM1 homodimerization	[14, 108]
S216	S217	Phosphorylation	Unknown	Blocks S-Adenosylmethionine (SAM) binding, Promotes CARM1 cytoplasmic localization	[13]
S447	S448	Phosphorylation	PKA	Facilitates CARM1 binding to estrogen receptor α (ERα)	[15]
S595	S595	Phosphorylation	p38 MAPK	Prevents the nuclear translocation of CARM1	[16]
T131 T138 T143	T132 T139 T144	Phosphorylation	GSK-3β LKB1	Maintains methyltransferase activity, Stabilizes CARM1 by impair its ubiquitination and degradation	[109]
R550	R551	Methylation	CARM1	Regulate transcription and splicing events	[8, 19]
S595, S598, T601 and T603	S595, S598, T601 and T603	O-GlcNAcylation	OGT	Determines substrate specificity	[21]
K470	K471	Ubiquitination	SKP2	Degrades CARM1 in the nucleus	[18]
S595	S595	Phosphorylation	JAK mutant JAK2^V617F^	Decreases activity	[16]
Unknown, likely multiple sites	Unknown, likely multiple sites	Polyubiquitination	CHIP ligase	Downregulation of PRMT5 through ubiquitin-mediated proteasomal degradation via K48-linked chains	[18, 110, 111]

Phosphorylation at specific serine (S) sites, such as S217, S229, S448, and S572, which are conserved among CARM1 from different species but not usually among all other PRMTs, has been identified as crucial regulator of CARM1 activity, localization, and stability. CARM1 crystal structures notably show that in the SAM binding cavity, the hydroxyl group of S217 forms a strong hydrogen bond with the carbonyl oxygen atom of tyrosine 153 (Y153). Phosphorylation of S217 breaks this hydrogen bond, eliminating SAM binding and methyltransferase activity while leaving dimerization or coactivator function unaffected. This phosphorylation event dynamically regulates CARM1 enzymatic activity throughout the cell cycle, peaking in mitosis and sharply decreasing to a basal level upon entry into the G1 phase [13]. Similarly, phosphorylation at S229 hampers CARM1 enzymatic activity by blocking SAM binding and abrogating homodimerization of CARM1 (Table 1). The introduction of a glutamic acid mutation at S229 (S229E), mimicking the phosphorylated serine, generates a dominant-negative CARM1 variant incapable of stimulating estrogen receptor (ER)-dependent gene expression [14]. Furthermore, phosphorylation of S448 by PKA mediates the direct interaction of CARM1 with the unliganded hormone-binding domain (HBD) of ERα, a crucial step in cAMP activation of ERa [15]. Additional phosphorylation events, such as S572 phosphorylation by p38MAPK, influence the subcellular localization of CARM1. Directly phosphorylation by p38MAPK at S572 (corresponding to S595 in CARM1 isoform 1) prevents CARM1 translocation to the nucleus [16].

Phosphorylation also crosstalk with other PTMs to regulate the stability of CARM1. GSK-3β-mediated phosphorylation at T131 stabilizes CARM1 by ablating its ubiquitination and subsequent protein degradation, thereby contributing to the impediment of lung epithelial cell migration under oxidative stress [17]. Under nutrient-rich conditions, the SKP2, an SCF E3 ubiquitin ligase, in the nucleus controls CARM1 stability. Nutrient deprivation triggers phosphorylation of FOXO3a by AMPK, leading to transcriptional repression of SKP2, and preventing CARM1 from being ubiquitinated and degraded [18].

An indispensable regulatory mechanism for CARM1 activity, responsive to diverse physiological cues, involves automethylation. Automethylation of CARM1 occurs at its own arginine residue R550 (R551 in mouse) in exon 15, which is conserved among all vertebrate CARM1 proteins. While this modification does not impact enzymatic activity, it is essential for proper protein-protein interactions of CARM1, especially in regulating transcription coactivator activity, substrate selectivity and alternative splicing events [8, 19]. CARM1 was previously recognized for O-GlcNAcylation by O-linked N-acetylglucosamine transferase (OGT) [20]. S595, S598, T601 and T603 in the C-terminus of CARM1 revealed potential O-GlcNAc addition. Interestingly, diminishing O-GlcNAcylation at these four sites did not affect the dimerization or protein stability of CARM1. However, it did dictate different substrate specificity, indicating the importance of O-GlcNAcylation for CARM1 substrate specificity [21]. OGT overexpression consistently prevents CARM1 phosphorylation by an upstream kinase, and methylation of downstream H3R17 [22]. Moreover, overexpression of OGT inhibits mitotic phosphorylation and alters the cellular localization of CARM1 [22], suggesting O-GlcNAcylation may be involved in controlling the localization of CARM1. Usually, CARM1 was enriched in the nucleus in interphase cells and concentrated in the pericentriolar area as DNA condensed and began to align on the metaphase plate mitosis. OGT overexpression renders the diffuse throughout the cell of CARM1 rather than localization to the pericentriolar area during mitosis [22].

### Altered demethylation by arginine demethylase

The dynamic arginine methylation level is controlled by the coordinated regulation of CARM1 and demethylase. Various reports have indicated that LSD1, the first discovered histone demethylase, binds to DNA on the nucleosome through the CoREST’s SANT2 domain, facilitating the demethylation of nucleosomal substrates [23]. Recent studies have revealed the coexistence of LSD1 and CARM1 within the same complex, exhibiting an essential co-activation role in the cAMP and ligand-dependent endoplasmic reticulum (ER) activation pathway [24]. Additionally, CARM1 dimethylated LSD1 at the R838 site, promoting the binding of the deubiquitinase USP7, while methylation of LSD1 R838 enhances its binding to the E-cadherin and vimentin promoters, leading to the demethylation of H3K4me2 and H3K9me2, respectively. However, the mechanism by which LSD1 mediates H3K4me1/2 and H3K9me1/2 demethylation remains unclear [25]. Meanwhile, JMJD6, an iron- and 2-oxoglutarate-dependent dioxygenase, catalyzes the demethylation of methylated arginine and lysine residues on histone and non-histone proteins. CARM1-mediated MED12 methylation is involved in the activation of ERα-specific enhancers. It has significantly reduced the interaction between MED12 and CARM1 without JMJD6 [26, 27]. According to a recent study, RDM activity is present in other 2OG-dependent JmjC oxygenase KDMs, such as KDM3A, KDM4E, KDM5C, and KDM6B [28]. It is important to note that further experimental research is required to elucidate the mutual interactions and regulatory mechanisms of CARM1-mediated methylation and demethylation by LSD1 and JMJD6 (Fig. 3c).

## The pathophysiologic function of CARM1 in carcinogenesis

It has been discovered that CARM1, an oncogenic protein, is overexpressed in a number of cancers, including breast [9, 29, 30], ovarian [31, 32], hematopoietic [33, 34], liver [35], pancreatic [36], colorectal [37, 38], prostate [39], bone [17], oral [40], lung [41], and melanoma [42]. However, recent studies suggests that CARM1 suppresses tumors in lung, liver, and pancreatic cancers as well [35, 36, 43]. Notably, the full-length CARM1 seems to exhibit tumor-suppressive function, while the short isoform (CARM1ΔE15) appears to have an oncogenic function in breast tumors [10]. These observations suggest a context-dependent function of CARM1 in cancer. Here, we discussed how CARM1 modulates various biological functions, including chromatin remodeling and gene activation, metabolism, autophagy, redox homeostasis, and signal transduction, promoting carcinogenesis (Table 2) [27, 44–46].

**Table 2:** Validated CARM1 substrates with their functions in tumorigenesis

Substrates	Methylated arginine residue (human)	Biological function	Impact of the methylation on the function of the substrate	Refs
*Transcriptional activation*				
AIB1	R849 R854 R1171 R1177 R1188	Transcriptional activation	Impairs association with CBP	[116, 117]
BAF155	R1064	Transcriptional activation	Switches promoter occupancy from BAF155 to EZH2	[32, 60]
CBP	R601 R625	Transcriptional activation	Disrupts the binding between KIX and the kinase-inducible domain of CREB, leading to inhibition of CREB activation	[118]
	R714 R742 R768 R2151	Transcriptional activation	Induces for GRIP-1- and steroid hormone-mediated gene activation; increases histone-acetyltransferase activity	[56, 119]
Histone H3	R2	Transcriptional activation	ND	[47, 120, 121]
	R17	Transcriptional activation	Transcriptional activation	[47, 48, 51, 52, 120–122]
	R26	Transcriptional activation	Transcriptional activation and repression	[47, 120, 123, 124]
	R42	Transcriptional activation	Transcriptional activation	[125]
HSP70	R469	Transcriptional activation	Controls the activation of the RARβ2 gene mediated by retinoic acid	[126]
MED12	R1782 R1792 R1854 R1859 R1862 R1871 R1899 R1910 R1912 R1994 R2015	Transcriptional activation	Suppresses p21 transcription Mediates interaction with TDRD3 Important for ER-α mediated gene transcription	[26, 27, 29, 93]
NFIB	R388	Transcriptional activation		[87]
p300	R580 R604 R651	Transcriptional activation	Prevents CREB activation by preventing KIX from attaching to the kinase-inducible domain of CREB.	[118]
	R754	Transcriptional activation	Important for binding to BRCA1	[45]
	R2142	Transcriptional activation	Impairs binding to GRIP1 and ACT	[127, 128]
LSD1	R838	Transcriptional activation	Affects stabilization	[25]
Pax7	R10 R13 R22 R37	Transcriptional activation	Induces *Myf5* expression	[16, 129]
pRb	R775 R787 R798	Cell cycle	Negatively regulates tumor suppressor function	[63]
YY1	R281 R294 R323 R342 R363 R381		Unknown	[40]
RUNX1	R223		Regulates binding to DPF2	[34]
NOTCH1	R2263 R2272 R2313 R2327 R2372		Controls stability	[83]
NOTCH2	R1786 R1838 R2047		Enhances its association with mastermind-like protein 1 (MAML1)	[130]
Sox2	R113	Transcriptional activation	Enhances self-association	[131]
*RNA processing*				
CA150	R28 R30 R41 R48	RNA processing	Allows interaction with the Tudor domain of SMN	[65]
HuD	R248	RNA processing	Affects mRNA turnover of p21cip1/waf1	[64]
p54nrb	R357 R365 R378	RNA processing	reduces binding to mRNAs containing IRAlus	[46]
PABP1	R455 R460 R506	RNA processing	No impact on stability or distribution	[112, 132, 133]
RNA Pol II	R1810		Facilitates expression of select small nuclear RNAs	[134]
CARM1	R550		Affects pre-mRNA splicing	[8, 19]
HuR	R217	RNA processing	Affects subcellular localization and stability	[135–137]
*Metabolism*				
MDH1	R248	Metabolism (Glutamine metabolism)	Inhibits activity	[36]
GAPDH	R234	Metabolism (Aerobic glycolysis)	Inhibits glycolysis	[35]
RPIA	R42	Metabolism (Pentose phosphate pathway)		[74]
PKM2	R445 R447 R455	Metabolism (Aerobic glycolysis)	Increases activity	[67, 68]
Others				
PRMT5	R505		Essential for oligomerization and methyltransferase activity	[49]
TARPP	R655		Exact function unknown	[138]

### Transcriptional coactivator

**Fig. 4:**
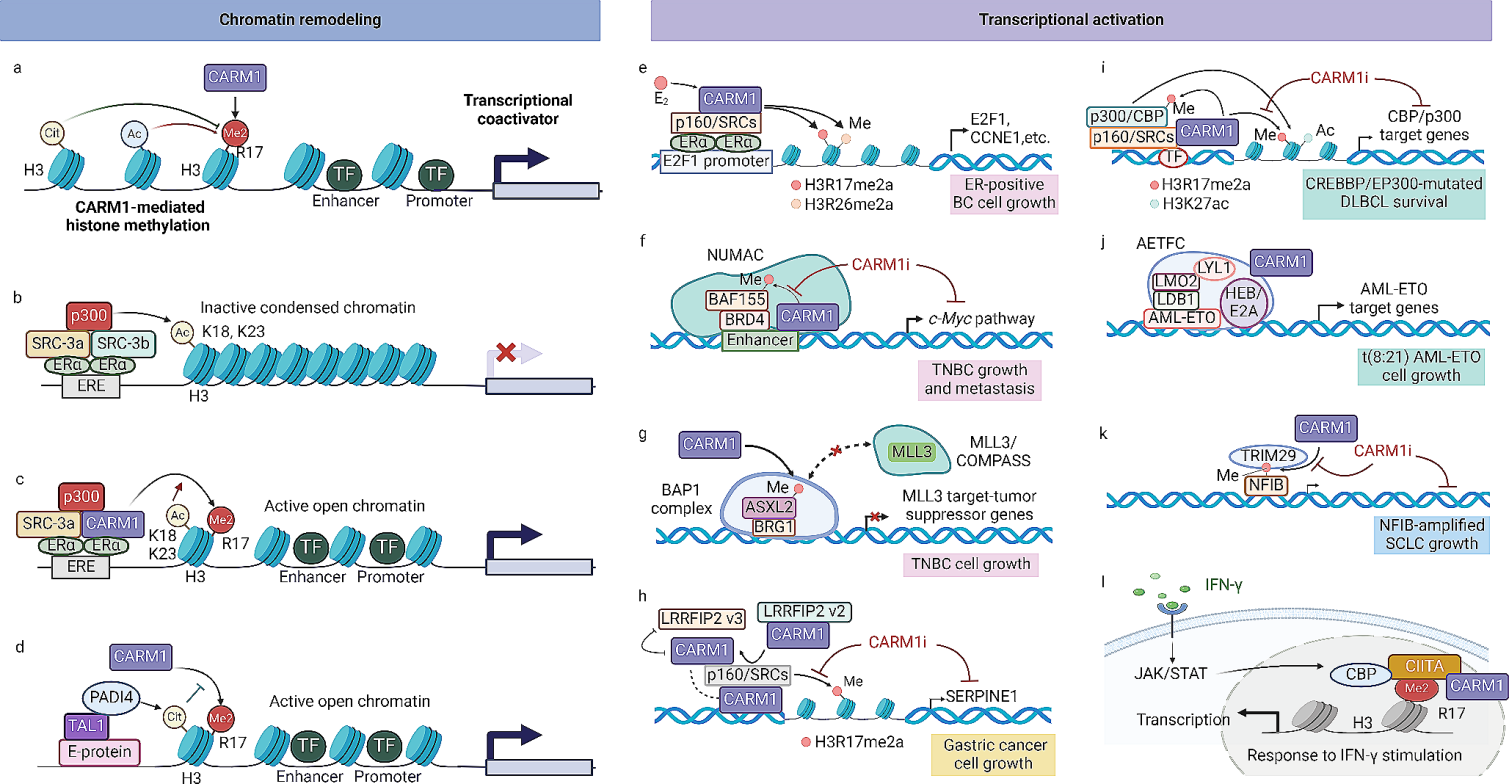
CARM1 controls the transcriptional activation in cancer. CARM1-mediated methylation works together with histone acetylation or citrullination to promote transcriptional activation by remodeling chromatin and releasing core repressors from chromatin (**a**, **b**). Preacetylation of histone H3 by p300 stimulates methylation of H3R17 by CARM1 (**c**), whereas citrullination of H3R17 by PADI4 blocks this process (**d**), creating active open chromatin for transcription. CARM1-mediated histone methylation serves as a platform for recruitment of transcriptional complexes, like p160 (**e**, **h**, **i**), NUMAC (**f**), BAP1 (**g**), AETFC (**j**). In addition, CARM1 modifies key regulatory factors in the transcription complex through methylation, promoting the recruitment of the transcriptional coactivator to enhancers. By promoting gene transcription, CARM1 has been implicated in the pathogenesis and poor prognostic outcomes of several cancers, such as breast cancer (**e**, **f**, **g**), gastric cancer (**h**), DLBCL (**i**, **j**), SCLC (**k**). CARM1 can also regulate gene transcription in response to extracellular IFN-γ signals (**l**). Abbreviations: ASXL2, ASXL Transcriptional Regulator 2; BRG1, brahma-regulated gene 1; CBP, CREB-binding protein; E2F1, E2F Transcription Factor 1; ERα, estrogen receptor alpha; ERE, estrogen response element; LYL1, the Lymphoblastic leukemia 1; NFIB, nuclear factor I B; MLL3, mixed-lineage leukemia 3; PADI4, peptidylarginine deiminase IV; SRC-3, steroid receptor coactivator-3; TAL1, T cell acute lymphocytic leukemia 1; TRIM29, tripartite motif containing 29

It has been proposed that CARM1 plays a crucial function in transcriptional activation by methylating histone H3, modifying chromatin remodeling and facilitating coactivator complex assembly (Fig. 4). CARM1 skillfully directs its coactivator activity in cancers by methylating histone H3 at R2, R17, and R26. Notably, via a random kinetic mechanism, CARM1 exhibits a preference for methylating H3R17 over H3R26 [47]. Surprisingly, the overall levels of H3R17me2a in CARM1 knockout mice did not significantly fall, as PRMT6 was found to compensate by depositing the H3R17me2a mark [48]. CARM1 extends its regulatory influence beyond H3 methylation. Consistently, CARM1-mediated methylation of PRMT5 at Arg505 negatively regulates the expression of the human γ-globin gene in erythroleukemia cells by decreasing H4R3me2s enrichment at its gene promoter [49]. Asymmetrically orchestrated methylation by CARM1 collaborates with histone acetylation to facilitate transcription activation through chromatin remodeling and discharging corepressors from chromatin [50]. In contrast to PRMT1’s preference for unacetylated histone tails in H4R5 methylation, CARM1 prefers methylating H3R17 in acetylated histone tails [51]. Preacetylation of histone H3 at lysine residues 18 and 23 by EP300 has been observed to stimulate the methylation of H3R17 by CARM1. Conversely, the citrullination of H3 at Arg-17 by PADI4 impedes this process. Notably, CARM1-mediated H3 arginine methylation protects its acetylation by displacing the nucleosome remodeling and deacetylase (NuRD) complex and TIF1 corepressors from chromatin [44].

Methylation of histone H3 by CARM1 has been associated with the activation of many oncogenic transcriptional factors. The recruitment of CARM1 to the pS2 gene, sometimes referred to as TFF1, is shown in response to estrogen (E2) and cAMP. Methylation of histone H3 by CARM1 has been associated with ER-target gene pS2 activation depending on the p160 proteins. H3R17me2a mediated by CARM1 recruits PAF1c, which functions as an arginine methyl histone effector, thereby facilitating the transcriptional activation of ERα targets [50]. CARM1-mediated H3R17me also regulates Yap1 and cell cycle signaling pathways, modulating mouse embryo development [52]. Though producing H3R17me, CARM1 interplays with CIITA and CBP during IFN-γ stimulation to induce gene activation [53]. CARM1-mediated H3R17 methylation promotes gene transcription and cell proliferation, which has been linked to the pathogenesis of tumors and unfavorable prognostic outcomes in several cancers, such as breast, lung, and liver cancers.

CARM1 also interacts with nuclear hormone receptors (NRs), regulating the transcription activation of oncogenic hormone signaling. CARM1 binds to the carboxyl-terminal region of the p160 coactivators, including SRC-1, GRIP1/TIF2, and p/CIP, which serve as a binding platform for p300/CBP and coiled-coiled coactivator in transcriptional activation by NRs [14, 54]. CARM1 mediates the methylation of multiple Arg residues within CBP, which prevents its binding to coactivators like CREB and p160 nuclear receptor coactivator GRIP1 [45, 53, 55]. The methylation of CBP by CARM1 led to the specific target gene hubs being identified by the gene-selective interaction of methylated CBP species recruited by estrogen with various HAT activity [56]. Inhibition of CARM1 can further decrease the HAT activity of CBP and the expression of target genes, leading to synthetic lethality in diffuse large B cell lymphoma (DLBCL) tumors with mutant CREBBP/EP300 [57]. Additionally, SREBP2-mediated transcriptional activation of mevalonate pathway genes is suppressed by CARM1-mediated CBP methylation at R601 and R672 [58]. Several transcriptional regulators were involved in the regulating role of CARM1 in ERa-regulated gene transcription. AIB1, whose methylation regulates its activity and stability, is necessary for CARM1 recruitment to ERa-regulated promoters [59]. CARM1 facilitates the recruitment of the MED12/JMJD6 mediator complex onto ERa-bound active enhancers by methylating MED12 at several arginine sites [26, 27]. Additionally, the expression of c-Myc pathway genes was affected by CARM1-mediated BAF155 methylation at R1064, as the methylation directs BAF155 to unique chromatin regions [60].

CARM1 promotes tumorigenesis by regulating the function of tumor suppressors like p53, pRb and BRCA1. CARM1 only interacted with the p53 C terminus (residues 300–393), highly dependent on residues 370–393 [61]. Similarly, deleting the p53 C-terminal region disrupted interactions with CARM1, leading to a noticeable reduction in H3R17 methylation. This deletion also hindered the recruitment of CARM1 to the p53 response element [61]. In vitro experiments, whether co-administered or sequentially applied, revealed that the three coactivators—PRMT1, p300, and CARM1—most significantly stimulated p53-mediated transcription. Within two hours of UV irradiation, the binding of p53 and p300 to the p53 response element reached its peak level, followed by the subsequent binding of CARM1 at the same site. Concurrently, H4R3 methylation, presumably by PRMT1, occurred alongside p300. Therefore, CARM1, PRMT1 and p300 cooperate to achieve the cooperative effects of histone acetylation and methylation sequentially, thereby modulating p53-mediated coactivators [61, 62]. These findings aligned with observations made regarding CARM1, PRMT1, p300, and p160 family coactivators in nuclear receptor-mediated transcription. Similarly, CARM1-mediated methylation of p300 protein at the Arg754 residue facilitates BRCA1 recruitment to the p53 binding area of the p21 promoter, thereby synergistically promoting p21 expression in response to DNA damage [45]. Additionally, CARM1 methylates pRb at R775, R787, and R798, disrupting the formation of E2F-1/DP1-pRb complex and inhibiting its repression of E2F-1 transcriptional activation [63].

### RNA processing

In addition to its role in transcriptional coactivation, CARM1 exerts regulatory control over RNA processing and noncoding RNAs (ncRNAs) via methylating various RNA-binding proteins (HuD and HuR), splicing factors (SAP49, CA150, SmB, and U1C), as well as RNA binding proteins such as poly-A-binding protein 1 (PABP1). For instance, CARM1’s methylation of HuD blocks p21cip1/waf1 mRNA from entering the decay route, sustaining PC12 cells proliferatively [64]. CA150 requires CARM1-mediated methylation to interact with the Tudor domain of SMN, offering a molecular explanation for the putative role of SMN in pre-mRNA splicing [65]. CARM1 activity is also essential for MED12 to activate ncRNAs. Methylation of MED12 at R1899 recruits the TDRD3/TOP3B complex, which has been shown to associate with enhancer RNAs (eRNAs) that correlate with enhancer-promoter looping and gene activation [26]. Moreover, MED12 methylation recruits p300 protein to acetylate histone H3K27, promoting eRNA transcription from activated enhancers. This eRNA regulates H3K4me3 at the S region and recruits DNA break and repair complex for class switching recombination (CSR) [66]. One CARM1 isoform preserving introns 15 and 16 binds to the U1 small nuclear RNP-specific protein U1C, influencing the selection of 5′ splice site in pre-mRNA splicing [12]. Additionally, CARM1 regulates nuclear retention of mRNAs containing IRAlus via two mechanisms: first, it methylates the p54nrb coiled-coil domain, reducing p54nrb binding to IRAlus mRNAs; second, CARM1 reduces paraspeckle formation by NEAT1 transcription. This effect is inhibited by suitable stimulation like poly(I: C) treatment, increasing NEAT1 production, unmethylated p54nrb, and nuclear retention of IRAlus mRNAs at paraspeckles [46].

### Metabolism

**Fig. 5:**
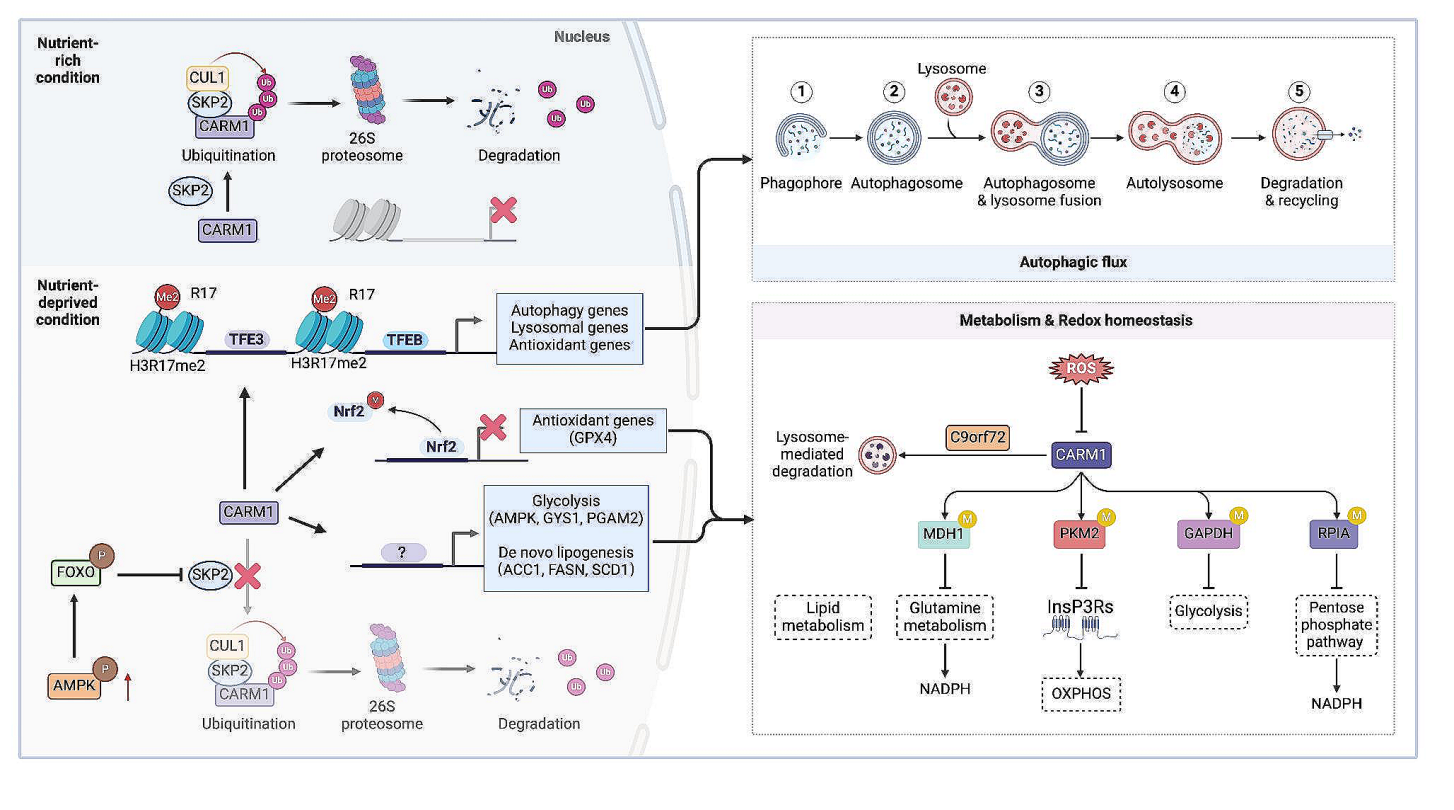
CARM1 controls autophagy, metabolism and redox homeostasis in tumors. In normal condition, the nuclear CARM1 was degraded by the UPS. During prolonged nutrient starvation, activated AMPK phosphorylates FOXO3, leading to the transcriptional repression of SKP2 and reduced degradation of CARM1. Elevated CARM1 increases H3R17me2, thereby activating the TFEB or TRE3-mediated autophagy and lysosomal gene transcription, which are crucial for developing resistance to chemotherapy. The methylation of Nrf2 by CARM1 limits its nuclear translocation and, subsequently, the transcription of GPX4, accelerating oxidative damage. CARM1 coordinates the transcriptional reprogramming of metabolic pathways, such as altering the expression AMPK and PGAM2 in glycolysis, as well as ACC1, SCD1 and FASN in de novo lipogenesis. CARM1 exhibits regulatory roles across various metabolic pathways, encompassing glutamine metabolism, glycolysis, OXPHOS, the pentose phosphate pathway, and lipid metabolism. The CARM1-dependent methylation of PKM2 reduces InsP3R expression, shifting metabolism from OXPHOS to aerobic glycolysis. In liver cancer cells, AMPK-dependent upregulation of CARM1 during glucose starvation inhibits glycolysis through GAPDH methylation. Methylation of MDH1 by CARM1 inhibits its activity, suppressing glutamine metabolism under normal conditions. Conversely, ROS-induced inhibition of CARM1 activates glutamine metabolism under stress conditions in PDAC. Additionally, CARM1 regulates the cellular redox homeostasis. CARM1-mediated metabolism also adapts tumor cells to variable oxidative stress conditions. Notably, the RPIA and MDH1 are methylated by CARM1, altering the production of NADPH and the cellular redox source. Abbreviations: UPS, ubiquitin-proteasome system; ACC1, acetyl coenzyme A carboxylase 1; CUL1, Cullin-1; FASN, fatty acid synthase; FOXO, Forkhead box-containing protein; GAPDH, Glyceraldehyde 3-phosphate dehydrogenase; InsP3Rs, inositol-1,4,5-trisphosphate receptors; MDH1, malate dehydrogenase 1; OXPHOS, oxidative phosphorylation; PGAM2, Phosphoglycerate mutase 2; PKM2, pyruvate kinase M2; ROS, reactive oxygen species; RPIA, Ribose 5-Phosphate Isomerase; SCD1, stearoyl-CoA desaturase 1; Skp2, S-phase kinase-associated protein 2; TFE3, Transcription factor E3; TFEB, Transcription factor EB

CARM1 is a crucial regulator of metabolic reprogramming in cancers, including glucose, glutamine, lipid, glycogen and one-carbon metabolism (Fig. 5). The increased enzymatic activity of PKM2 aids glycolysis due to CARM1-mediated methylation at R445/447 [67]. Another study has demonstrated that CARM1-mediated methylation at R445/447/455 of PKM2 impedes mitochondrial oxidative phosphorylation (OXPHOS). Methylated PKM2 suppresses the expression of inositol 1, 4, 5-trisphosphate receptors (IP3Rs) in mitochondria, curtailing calcium influx from the endoplasmic reticulum (ER) [68].

CARM1 induces an up-regulation of glycolytic flux by methylating PPP1CA at R23, which enhances the activities of PFK-1 and PFKFB. Similarly, by governing the expression of PDK3, CARM1 also diminishes OXPHOS flux and the tricarboxylic acid cycle (TCA) [69]. Consequently, CARM1 shifts the metabolism balance from OXPHOS to aerobic glycolysis, fostering tumor cell proliferation, migration and metastasis. Additionally, the expression of CARM1 affects how cells react when faced with nutritional shortages. When exposed to extracellular serine limitation, cells lacking the *Carm1* gene exhibit significant survival advantages over wild-type cells due to their reduced PKM2 activity redirects glucose flux toward the de novo serine biosynthesis, promoting cell proliferation without extracellular serine [67]. Glucose starvation raises CARM1 protein levels, which inhibits the catalytic activity of GAPDH for glycolysis by methylating R234, slowing tumor cell proliferation [35].

Similarly to its role in glucose metabolism, CARM1 regulates glutamine metabolism by methylation of vital metabolic enzymes. CARM1 mediates the methylation of MDH1 at R248, disrupting its dimerization and inhibiting catalytic activity. MDH1 is essential for glutamine-dependent synthesis of NADPH, which contributes to the redox homeostasis of PDAC cells. Repression of MDH1 by CARM1 dampens mitochondria respiration and glutamine metabolism. To cope with oxidative stress, oncogenic activating KRAS mutation or oxidative stress in PDAC suppresses CARM1-mediated MDH1, rewiring glutamine metabolism to support NADPH production and protecting PDAC cells from oxidative damage [36].

CARM1 also orchestrates the transcriptional reprogramming of fatty acid metabolism to promote ovarian cancer formation. Genes like ACC1 and FASN that encode rate-limiting enzymes involved in de novo fatty acid metabolism are expressed more frequently. Moreover, CARM1 upregulates stearoyl-CoA desaturase 1 (SCD1), an enzyme that desaturates fatty acids to generate monounsaturated fatty acids. The enhanced *de novo* fatty acid synthesis and subsequent synthesis of monounsaturated fatty acids contribute to tumorigenesis [70]. CARM1 is also necessary for regulating lipid metabolism under nutrient stress caused by glucose deprivation. Through lysosomal degradation, C9orf72 adversely affects the amount of CARM1 protein levels. Loss of C9orf72 causes CARM1 to become more enriched in the nucleus and more active as an epigenetic activator, linking to ACC promoter regions, causing overexpression of lipid metabolism genes during fasting [71]. Moreover, methyltransferase-deficient CARM1 mutants, such as CARM1-VLD and CARM1-E267Q, severely repress the expression of AMPK and PGAM2. Therefore, CARM1 expression and associated methyltransferase activity are essential for expressing genes implicated in glycogen metabolism and human glycogen storage disorders [72].

### Redox homeostasis

Redox homeostasis is imperative for the physiological functions of normal cells and the pursuit of cancer cells. CARM1 emerges as a crucial regulator in controlling oxidative stress (Fig. 5). Its interaction with Nrf2 confines the methylation, which limits Nrf2 nuclear translocation and subsequent transcription of GPX4. The suppression of Nrf2/GPX4 signaling by CARM1 overexpression accelerates doxorubicin-induced ferroptosis in cardiomyocytes [73]. CARM1 also orchestrates remodeling of the metabolic network in cancer cells, adapting them to variable oxidative stress conditions. By inducing R42 methylation, CARM1 increases the catalytic activity of ribose-5-phosphate isomerase A (RPIA), an enzyme in the pentose phosphate pathway (PPP). Under glucose deprivation, hypermethylation of RPIA by CARM1 amplifies oxidative PPP flux and NADPH production, augmenting colorectal cancer cell survival during glucose starvation [74].

Conversely, CARM1 also assumes a negative regulatory role in NADPH production through unconventional glutamine metabolism in PDAC cells. The arginine methylation of MDH1 by CARM1 inhibits glutamine metabolism and NADPH production, rendering PDAC cells more vulnerable to oxidative stress and suppressing cell proliferation. Notably, oxidative stress inhibits CARM1 activity and downstream MDH1 methylation. This remarkable finding suggests that CARM1 could act as a ROS sensor, influencing cancer’s redox balance and metabolism [36]. Therefore, although the mechanisms and effects warrant further investigation in distinct contexts, CARM1 is a critical regulator for fine-tuning redox homeostasis by modulating the antioxidant Nrf2 signaling and NADPH production.

### Autophagy

Autophagy is a highly conserved mechanism of self-digestion that promotes tumor development, survival, and chemoresistance in addition to preserving cellular viability and homeostasis in response to food shortage. The intricate regulation of autophagy at the molecular level involves CARM1-dependent histone arginine methylation (Fig. 5). Under normal/nutrient-rich conditions, CARM1 undergoes degradation by SKP2-mediated ubiquitin-proteasome system (UPS) in the nucleus or by C9orf72-dependent lysosomal proteolysis in cytosolic. During glucose starvation, CARM1 accumulation in the nucleus is facilitated by the AMPK/FOXO3a axis, which transcriptionally represses SKP2. The heightened levels of CARM1 exert a transcriptional coactivator for TFEB by methylating H3R17me2a, triggering the expression of several autophagy and lysosomal target genes [18, 75, 76]. Additionally, CARM1-mediated methylation of Pontin, a chromatin-remodeling factor, at R333 and R339 under glucose starvation serves as a scaffold for the recruitment of the histone acetyltransferase Tip60. This recruitment enhances H4 acetylation, subsequently activating autophagy gene transcription by FOXO3a. Interestingly, the CARM1-Pontin-FOXO3a signaling axis operates in the distal areas, utilizing enhancer activation to maintain autophagy gene expression and autophagic flux under glucose starvation [77]. CARM1 also encourages the nuclear translocation of TFE3, which often shares regulatory signaling networks with TFEB. This translocation induces autophagy through the nuclear AMPK-CARM1-TFE3 and cytoplasmic AMPK-mTOR signaling pathways [78, 79]. Through regulating autophagy, CARM1 accelerates the G1-S transition, promotes cancer cell growth, and mitigates ER stress-induced apoptosis [79].

### Inflammation

CARM1 participates in the ongoing inflammatory response, which is crucial to the malignant transformation, cancer propagation and metastasis. CARM1 functions as a transcriptional coactivator of NF-κB, promoting NF-κB recruitment to chromatin. In response to TNFα or LPS stimulation, NF-κB-dependent gene subsets are expressed less frequently in CARM1 deletion cells [80, 81]. It has also been mentioned that CARM1 and P300 work cooperatively to promote the NF-κB recruitment to chromatin and regulate the transcriptional control of inflammation-related genes during monocyte inflammatory stimulation [80]. Moreover, CARM1 modulates the NF-κB pathway by methylating its upstream regulators, like the Notch intracellular domain (NICD), which has been shown to interact directly with the NF-κB subunit. Through methylation at five conserved arginine residues in its C-terminal trans-activation domain, CARM1 controls the stability of the NICD protein. Crucially, the methylation may enhance the transcriptional action of Notch, implying that CARM1-mediated Notch methylation may also contribute to the inflammation of Notch-dependent illnesses like leukemia [82, 83].

## CARM1 serves as a therapeutic target and predictive biomarker of treatment response

Neoplasms dependent on CARM1 offer a novel avenue for anticancer interventions and serve as a prospective prognostic indicator for tumor advancement, metastatic potential, and resistance to therapeutic interventions. Small-molecule inhibitors and cell-penetrating peptides (CPP) have proven efficacious in selectively targeting CARM1 in cancer therapeutics, potentially overcoming intrinsic or acquired resistance to treatment. Moreover, CARM1’s anticipatory capacity in predicting treatment responsiveness establishes a foundation for developing more efficacious therapeutic strategies.

### CARM1 as a potential therapeutic target for cancers

CARM1 is a therapeutic target for otherwise untargetable transcription factors due to its regulating function in transcriptional activation. In AML, CARM1 promotes cell survival by interacting with the AE-containing AETFC complex, facilitating target gene expression [84]. CARM1 deletion prevents AML driven by oncogenic transcription factors without impacting normal hematopoiesis [85]. Overexpressed CARM1 in human grade-III breast tumors and PDAC promotes cancer cell proliferation by activating ERα or androgen receptor (AR) activity, respectively [43]. Targeting CARM1 may benefit ERα-positive breast cancer, given its specific recruitment to ERα-bound active enhancers and its requirement for estrogen/ERα-triggered transcriptional activation [29]. CARM1 collaborates with transcriptional coactivators like GRIP1 and CBP to regulate AR function. Its distinctive transcriptional regulatory mechanism, involving methylation of histones and other proteins in the transcription start complex, offers a targeted treatment option for prostate cancer, especially in androgen-independent cases [29, 86]. The methylation of NFIB by CARM1 is pivotal in small-cell lung cancer development. Disrupting CARM1 or inhibiting NFIB methylation decelerates the growth of small cell lung cancer cells [87].

Highly expressed CARM1 in malignancies promotes tumor metastatic ability through transcriptional regulation of oncogenic pathways, metabolic rewiring, and modulation of autophagy. The methylation of epigenetic regulatory proteins, including SWI/SNF core subunit BAF155, transcription factor NFIB, and histone lysine demethylase LSD1, is a notable mechanism of CARM1 in transcriptional activation of oncogenic pathways. Methylation of BAF155 guides it to specific chromatin regions, where it activates the c-Myc pathway and promotes tumor metastasis [88]. TNBC metastasis is further encouraged by CARM1-catalyzed methylation of BAF155, which recruits BRD4 to activate super-enhancer-addicted oncogenes and suppress the host immune response [89]. CARM1 stabilizes histone lysine demethylase LSD1 via USP7-dependent deubiquitination, promoting breast cancer cell motility and invasion [25]. Inhibition of CARM1 represses SERPINE1 expression, reducing the invasiveness of gastric cancer cells [90]. Besides transcriptional control, CARM1 regulates cellular metabolism, supporting tumor metastasis by switching OXPHAS to aerobic glycolysis via methylating the dimeric form of PKM2 at R445/447/455 [68]. CARM1 also contributes to the spread of metastatic disease through autophagy, as its suppression induces autophagy, preventing migration or invasion in NSCLC [91]. The CARM1 inhibitor SKI-73 reduces invasiveness by changing epigenetic plasticity [92]. Understanding the CARM1-addiction mechanism of cancer metastasis helps develop therapeutic strategies targeting this process.

### CARM1 serves as a potential prognostic marker for cancer therapeutic resistance

Overexpression of CARM1 in multiple cancers is closely associated with the development of therapeutic resistance. CARM1 methylates MED12 at R1862 and R1912, and mutations at these sites lead to resistance to chemotherapeutic agents, serving as a predictive sensor for how human malignancies will respond to commonly used chemotherapy drugs [93]. JMJD6 is reported to regulate the interaction between MED12 and CARM1, acting as a crucial regulator of breast cancer cell potential and a potential therapeutic target a possible target for treatment in cases of ER-positive breast cancer [27]. Sustained PKA activity results in the phosphorylation of CARM1, leading to tamoxifen resistance in breast tumors. This phosphorylation of CARM1 is necessary and sufficient for directly binding to the unliganded hormone-binding domain (HBD) of ERα. Furthermore, cAMP activation of ERα relies on this interaction [15].

Long-term exposure to chemotherapy may induce autophagy through epigenetic regulation, specifically by transcriptionally modifying TFE3 and TFEB coactivators, such as CARM1 [94]. Additionally, AMPK activates TFEB by increasing the levels of CARM1, which is implicated in chemotherapy resistance. The AMPK inhibitor SBI-0206965 sensitized tumor cells to doxorubicin, indicating a potential strategy to overcome chemotherapy resistance by inhibiting the AMPK-CARM1-TFEB axis-mediated autophagy [18, 95]. Heightened expressions of TFEB and Beclin-1 correlate with reduced survival in chemotherapy-treated invasive breast cancer patients. Positive correlations among CARM1, TFEB, SIRT1, and Beclin-1 in breast cancer suggest CARM1’s potential prognostic value and its role as a novel target to combat inherent or acquired drug resistance in breast cancer [96]. CARM1 also contributes to immunotherapy resistance. In a CRISPR/Cas9 screen using B16F10 melanoma cells, CARM1 has been identified as a negative regulator of T cell survival and proliferation. Inhibition of CARM1 supports the survival of tumor-infiltrating memory-like T cells and improves the antitumor activities of CD8^+^ T cells. Meanwhile, B16F10 cells are vulnerable to immunological assault by T cells and monotherapy with checkpoint antibodies (CTLA4 and DP-1) when CARM1 is knocked down or treated with CARM1 inhibitors [97]. Overall, CARM1 emerges as a crucial factor leading to the development of tumor therapeutic resistance, providing new insights into the potential development of cancer drugs.

### Targeting CARM1 for cancer treatment

With the advancement in strategies for determining the enzymatic activity and substrates of CARM1 (Table 3), different CARM1 inhibitors (CARM1i) have been developed with diverse molecular scaffolds and ways of interaction with CARM1 [3]. The CARM1i shows promise in the preclinical stage for treating various cancers [3]. Notable examples include EZM2302(3) and TP-064, which were developed to occupy the peptide substrate-binding pocket of CARM1 and exhibited antitumor effects against cancers such as breast cancer, multiple myeloma (MM), AML, and diffuse large B-cell lymphoma [57, 85, 92, 98, 99]. SKI-73 inhibits CARM1 by engaging the SAM-binding site, suppressing invasion but not proliferation of breast cancer cells [92]. Compound 43, a selective and potent CARM1 inhibitor, significantly affects solid tumors’ in vivo behavior. Its stable metabolic profile allows oral administration, presenting a viable treatment option for malignant solid cancers. Compound 43 has also been shown to increase the quantity of dendritic cells (DCs) and activated CD8^+^ T cells [100]. Additionally, seven compounds with new scaffolds specifically inhibiting CARM1 methyltransferase activity have been identified and confirmed through virtual screening. Compound NO.2 was selected for structural optimization due to its significant toxicity to breast cancer cells [101]. The oral administration of compound 49, based on a tetrahydroisoquinoline scaffold, demonstrates good antitumor activity in an AML xenograft model, showing high selectivity and intermediate pharmacokinetic characteristics [102].

**Table 3:** Assays for determining the enzymatic activity and substrates of CARM1

Methods	Principles	Advantages	Disadvantages	Refs
*Assays for determining the enzymatic activity of CARM1*				
Filter- or flash plate-based radio assays	Utilizing 3 H-SAM for the production of 3 H-methylated products	Precise, user-friendly, suitable for automation, and excellent reproducibility	Inconvenience	[112]
ELISA	Anti-methylarginine antibody	High specificity	Limited effectiveness due to antibody selectivity	[112]
Fluorescence immunoassay-based methyltransferase assay	Using fluorescence immunoassay to detect adenosine monophosphate (AMP), a highly targeted for histone methyltransferase (HMT)	High sensitivity, high reliability		[113]
FRC based on Forster resonance energy transfer (FRET)	Changes in the coumarin/fluorescein fluorescence ratio of FRCS in the presence of trypsin	Simple, quantitative, non-radiative		[114]
*Assays for determining the substrates of CARM1*				
In vitro methylation assays	Utilizing mass spectrometry (MS) to identify the methylation residues or autoradiography to detect the methylated proteins			[115]
In vivo methylation assays	Implementing diverse methods in conjunction with MS analysis. Cells underwent metabolic labeling using radioactive methionine and were subjected to immunoprecipitation for potential substrates. Alternatively, methylated peptides were detected through immunoprecipitation using pan-methylated antibodies and subsequent MS analysis			[115]

Recently, proteolysis-targeting chimera (PROTAC) strategies has been developed by harnessing the ubiquitin-proteasome system to destroy a target protein [103]. Compound 3b represents the creation of CARM1 PROTAC, a degrader that includes the CARM1 inhibitor TP-064, linker, and VHL E3 ligase ligand. It is anticipated that CARM1 PROTACs will be developed as a therapeutic drug to target CARM1-driven cancer, as this is the first development of effective and selective CARM1 PROTACs that achieve a degradation agent concentration 100 times less than the inhibitor [104].

Contrary to its abundance in other tumors, CARM1 exhibits low expression levels in liver cancer and PDAC. Therefore, activating CARM1 may be considered for therapeutic interventions against liver cancer and PDAC. In contrast to inhibitory drugs, CARM1 protein can also be introduced into donor cells to supply CARM1. The delivery of CARM1 protein to human mesenchymal stem cells (hMSCs) using CPPs enhances embryonic development, providing a valuable tool for improving mammalian embryo implantation through the regulation of histone methylation and gene expression [105]. Similarly, biologically active CARM1 protein delivered through a CPP induces chromatin remodeling via histone methylation in hMSCs. The differentiation efficiency of BM-hMSCs and AD-hMSCs into adipogenic, osteogenic, and myogenic cell lineages is greatly enhanced in vitro by CPP-CARM1 protein, indicating possible clinical uses for cancer treatment [106].

CARM1 has inherent therapeutic potential as a therapeutic target and serves as a biomarker for cells susceptible to synthetic lethality-based therapies. Its role in methylating BAF155 to regulate the antagonistic relationship between EZH2 and SWI/SNF indicates that EZH2 inhibition significantly retards the growth of ovarian cancers expressing CARM1, while having no impact on tumors lacking CARM1 [32]. By upregulating MAD2L2, EZH2 inhibition decreases DNA end resection in a CARM1-dependent manner, making CARM1-high, HR-proficient epithelial ovarian cancer cells more susceptible to the combined effects of EZH2 and PARP inhibitors [107]. This result suggests that EZH2 pharmacological inhibition provides a novel treatment strategy for cancers expressing CARM1. Additionally, CARM1 regulates the expression of target genes for the IRE1α/XBP1s pathway, which causes ovarian cancer cells overexpressing CARM1 to be selectively sensitive to the blockage of the IRE1α/XBP1s pathway. A significant synergistic impact is observed when treating DLBCLs without CBP/EP300 mutations with the combination of CBP/p300 and CARM1 inhibitors. In DLBCL cells, CARM1 inhibition mechanistically reduces CBP’s histone acetyltransferase (HAT) activity, downregulating its target genes and causing synthetic lethality [57].

## Conclusion and perspective

CARM1 is a crucial regulator of gene expression with an increasingly important role in tumorigenesis. On the one hand, CARM1 is overexpressed or overactivated in tumor cells; on the other hand, various oncogenic signals regulate the activity and localization of CARM1 by mediating specific PTMs. CARM1 mediates methylation modification of substrates, leading to tumor progression, metastasis, and drug resistance. This critical role of CARM1 in tumorigenesis makes it a new perspective in cancer drug development and a potential sensor for predicting the responsiveness of human cancers to commonly used chemotherapeutic drugs, providing prospects for therapeutic targets. Studying the mechanism of action of CARM1 in various cancer types can deepen our understanding of tumorigenesis and provide new insights and methods for early cancer diagnosis and treatment. Although recent findings have revealed new facets of CARM1 function, further studies are imperative to elucidate many unresolved aspects.

Presently, the comprehensive structural characterization of CARM1 remains incomplete. Existing knowledge suggests that the PH domain of CARM1 interacts with various proteins and several lncRNAs. Exploring CARM1’s interactions with other nucleic acids and delineating the interplay between proteins and nucleic acids, particularly in the context of binding PH-like domains, may unveil novel functionalities of CARM1. Moreover, there is current lack of preclinical investigations into the impact of PTMs on CARM1, leaving uncertainties regarding the specific functions influenced by these modifications. PTMs typically unfold sequentially, intricately constructing the accurate “PTM code” on the protein surface. Future efforts should aim to deepen our understanding of the intricate crosstalk between different PTMs and their collective influence on CARM1. While nuclear CARM1 had been identified in various speckles, the precise nature of these speckles requires further elucidation. Investigating the localization of CARM1 within other nuclear structures and discerning its potential functions in the cytoplasmic milieu are also avenues that demand thorough exploration. Additionally, CARM1 activity results in an augmented consumption of SAM as a methyl donor, leading to reduced SAM levels in rapidly growing cancer cells. Considering that methylation of histone, DNA, and mRNA is highly sensitive to SAM levels, the excessive SAM consumption associated with overactivated CARM1 may play a role in tumorigenesis by influencing epigenetic modifications. However, the precise mechanism underlying this involvement warrants further investigation.

CARM1 has emerged as a compelling therapeutic target across various cancers, with factors and pathways under its regulation exhibiting potential influence in diverse cancer types. Recent investigations have primarily focused on uncovering the role of CARM1 in cancer initiation and the underlying mechanisms driving tumorigenesis. Notably, the functional role of CARM1 manifests variability even among distinct subtypes of breast cancer. CARM1 is crucial in essential biological processes, including tumor cell proliferation, invasion, and metastasis. Inhibiting CARM1 activity has effectively restrained tumor cell growth and metastatic potential.

Furthermore, CARM1 interplays with several signaling pathways implicated in tumorigenesis, contributing to tumor initiation and progression through diverse routes. Consequently, targeting CARM1 holds promise as a novel approach for treating certain cancers. Encouragingly, there have been successful developments in CARM1-selective inhibitors, underscoring the feasibility of CARM1 as a therapeutic target in cancer therapy. Despite these advancements, a comprehensive understanding of the specific functions of CARM1 necessitates further in-depth exploration. Ongoing research efforts aim to elucidate the nuanced intricacies of CARM1’s roles and functions in the context of different cancer types.

## Data Availability

No datasets were generated or analysed during the current study.

## References

[1] Y. Chen et al., The role of histone methylation in the development of digestive cancers: a potential direction for cancer management. Signal. Transduct. Target. Ther. **5**(1), 143 (2020) 32747629 10.1038/s41392-020-00252-1 PMC7398912

[2] Q. Wu et al., Protein arginine methylation: from enigmatic functions to therapeutic targeting. Nat. Rev. Drug Discov. **20**(7), 509–530 (2021) 33742187 10.1038/s41573-021-00159-8

[3] W. Jin et al., Unraveling the complexity of histone-arginine methyltransferase CARM1 in cancer: from underlying mechanisms to targeted therapeutics. Biochim. Biophys. Acta Rev. Cancer. **1878**(4), 188916 (2023) 37196782 10.1016/j.bbcan.2023.188916

[4] K. Wang et al., PHGDH arginine methylation by PRMT1 promotes serine synthesis and represents a therapeutic vulnerability in hepatocellular carcinoma. Nat. Commun. **14**(1), 1011 (2023) 36823188 10.1038/s41467-023-36708-5 PMC9950448

[5] M. Santos, J.W. Hwang, M.T. Bedford, CARM1 arginine methyltransferase as a therapeutic target for cancer. J. Biol. Chem. **299**(9), 105124 (2023) 37536629 10.1016/j.jbc.2023.105124 PMC10474102

[6] S.K. Tewary, Y.G. Zheng, M.C. Ho, Protein arginine methyltransferases: insights into the enzyme structure and mechanism at the atomic level. Cell. Mol. Life Sci. **76**(15), 2917–2932 (2019) 31123777 10.1007/s00018-019-03145-x PMC6741777

[7] N. Troffer-Charlier et al., Functional insights from structures of coactivator-associated arginine methyltransferase 1 domains. Embo j. **26**(20), 4391–4401 (2007) 17882262 10.1038/sj.emboj.7601855 PMC2034665

[8] L. Wang et al., CARM1 automethylation is controlled at the level of alternative splicing. Nucleic Acids Res. **41**(14), 6870–6880 (2013) 23723242 10.1093/nar/gkt415 PMC3737532

[9] M.B. Davis et al., Expression and sub-cellular localization of an epigenetic regulator, co-activator arginine methyltransferase 1 (CARM1), is associated with specific breast cancer subtypes and ethnicity. Mol. Cancer. **12**(1), 40 (2013) 23663560 10.1186/1476-4598-12-40 PMC3663705

[10] D. Shlensky et al., Differential CARM1 isoform expression in subcellular compartments and among malignant and benign breast tumors. PLoS One. **10**(6), e0128143 (2015) 26030442 10.1371/journal.pone.0128143 PMC4451767

[11] M. Zheng et al., ESRP1 regulates alternative splicing of CARM1 to sensitize small cell lung cancer cells to chemotherapy by inhibiting TGF-β/Smad signaling. Aging (Albany NY). **13**(3), 3554–3572 (2021) 33495408 10.18632/aging.202295 PMC7906186

[12] N. Ohkura et al., Coactivator-associated arginine methyltransferase 1, CARM1, affects pre-mRNA splicing in an isoform-specific manner. J. Biol. Chem. **280**(32), 28927–28935 (2005) 15944154 10.1074/jbc.M502173200

[13] Q. Feng et al., Biochemical control of CARM1 enzymatic activity by phosphorylation. J. Biol. Chem. **284**(52), 36167–36174 (2009) 19843527 10.1074/jbc.M109.065524 PMC2794732

[14] K. Higashimoto et al., Phosphorylation-mediated inactivation of coactivator-associated arginine methyltransferase 1. Proc. Natl. Acad. Sci. U S A **104**(30), 12318–12323 (2007) 17640894 10.1073/pnas.0610792104 PMC1941467

[15] S. Carascossa et al., CARM1 mediates the ligand-independent and tamoxifen-resistant activation of the estrogen receptor alpha by cAMP. Genes Dev. **24**(7), 708–719 (2010) 20360387 10.1101/gad.568410 PMC2849127

[16] N.C. Chang et al., The Dystrophin Glycoprotein Complex regulates the epigenetic activation of muscle stem cell commitment. Cell. Stem Cell. **22**(5), 755–768e6 (2018) 29681515 10.1016/j.stem.2018.03.022 PMC5935555

[17] S. Li et al., The overexpression of CARM1 promotes human Osteosarcoma Cell Proliferation through the pGSK3β/β-Catenin/cyclinD1 signaling pathway. Int. J. Biol. Sci. **13**(8), 976–984 (2017) 28924379 10.7150/ijbs.19191 PMC5599903

[18] H.J. Shin et al., AMPK-SKP2-CARM1 signalling cascade in transcriptional regulation of autophagy. Nature. **534**(7608), 553–557 (2016) 27309807 10.1038/nature18014 PMC5568428

[19] P. Kuhn et al., Automethylation of CARM1 allows coupling of transcription and mRNA splicing. Nucleic Acids Res. **39**(7), 2717–2726 (2011) 21138967 10.1093/nar/gkq1246 PMC3074151

[20] W.D. Cheung et al., O-linked beta-N-acetylglucosaminyltransferase substrate specificity is regulated by myosin phosphatase targeting and other interacting proteins. J. Biol. Chem. **283**(49), 33935–33941 (2008) 18840611 10.1074/jbc.M806199200 PMC2590692

[21] P. Charoensuksai et al., O-GlcNAcylation of co-activator-associated arginine methyltransferase 1 regulates its protein substrate specificity. Biochem. J. **466**(3), 587–599 (2015) 25585345 10.1042/BJ20141072

[22] K. Sakabe, G.W. Hart, O-GlcNAc transferase regulates mitotic chromatin dynamics. J. Biol. Chem. **285**(45), 34460–34468 (2010) 20805223 10.1074/jbc.M110.158170 PMC2966060

[23] M. Yang et al., Structural basis for CoREST-dependent demethylation of nucleosomes by the human LSD1 histone demethylase. Mol. Cell. **23**(3), 377–387 (2006) 16885027 10.1016/j.molcel.2006.07.012

[24] M.A. Bennesch et al., LSD1 engages a corepressor complex for the activation of the estrogen receptor α by estrogen and cAMP. Nucleic Acids Res. **44**(18), 8655–8670 (2016) 27325688 10.1093/nar/gkw522 PMC5062963

[25] J. Liu et al., Arginine methylation-dependent LSD1 stability promotes invasion and metastasis of breast cancer. EMBO Rep. **21**(2), e48597 (2020) 31833203 10.15252/embr.201948597 PMC7001506

[26] D. Cheng et al., CARM1 methylates MED12 to regulate its RNA-binding ability. Life Sci. Alliance. **1**(5), e201800117 (2018) 30456381 10.26508/lsa.201800117 PMC6238599

[27] W.W. Gao et al., JMJD6 licenses ERα-Dependent enhancer and coding gene activation by modulating the recruitment of the CARM1/MED12 co-activator complex. Mol. Cell. **70**(2), 340–357e8 (2018) 29628309 10.1016/j.molcel.2018.03.006 PMC6258263

[28] S.T. Williams et al., Studies on the catalytic domains of multiple JmjC oxygenases using peptide substrates. Epigenetics. **9**(12), 1596–1603 (2014) 25625844 10.4161/15592294.2014.983381 PMC4623018

[29] B.L. Peng et al., A hypermethylation strategy utilized by enhancer-bound CARM1 to promote estrogen receptor α-dependent transcriptional activation and breast carcinogenesis. Theranostics. **10**(8), 3451–3473 (2020) 32206101 10.7150/thno.39241 PMC7069091

[30] H. Cheng et al., Overexpression of CARM1 in breast cancer is correlated with poorly characterized clinicopathologic parameters and molecular subtypes. Diagn. Pathol. **8**, 129 (2013) 23915145 10.1186/1746-1596-8-129 PMC3766166

[31] N. Nakayama et al., Cancer-related transcription regulator protein NAC1 forms a protein complex with CARM1 for ovarian cancer progression. Oncotarget. **9**(47), 28408–28420 (2018) 29983869 10.18632/oncotarget.25400 PMC6033357

[32] S. Karakashev et al., CARM1-expressing ovarian cancer depends on the histone methyltransferase EZH2 activity. Nat. Commun. **9**(1), 631 (2018) 29434212 10.1038/s41467-018-03031-3 PMC5809368

[33] S. Leonard et al., Arginine methyltransferases are regulated by Epstein-Barr Virus in B cells and are differentially expressed in Hodgkin’s lymphoma. Pathogens. **1**(1), 52–64 (2012) 25436604 10.3390/pathogens1010052 PMC4235682

[34] L.P. Vu et al., PRMT4 blocks myeloid differentiation by assembling a methyl-RUNX1-dependent repressor complex. Cell. Rep. **5**(6), 1625–1638 (2013) 24332853 10.1016/j.celrep.2013.11.025 PMC4073674

[35] X.Y. Zhong et al., CARM1 methylates GAPDH to regulate glucose metabolism and is suppressed in Liver Cancer. Cell. Rep. **24**(12), 3207–3223 (2018) 30232003 10.1016/j.celrep.2018.08.066

[36] Y.P. Wang et al., Arginine methylation of MDH1 by CARM1 inhibits glutamine metabolism and suppresses pancreatic Cancer. Mol. Cell. **64**(4), 673–687 (2016) 27840030 10.1016/j.molcel.2016.09.028

[37] Y.R. Kim et al., Differential CARM1 expression in prostate and colorectal cancers. BMC Cancer. **10**, 197 (2010) 20462455 10.1186/1471-2407-10-197 PMC2881889

[38] M. Zhang et al., Coactivator-associated arginine methyltransferase 1 promotes cell growth and is targeted by microRNA-195-5p in human colorectal cancer. Tumour Biol. **39**(3), 1010428317694305 (2017) 28345460 10.1177/1010428317694305

[39] H. Hong et al., Aberrant expression of CARM1, a transcriptional coactivator of androgen receptor, in the development of prostate carcinoma and androgen-independent status. Cancer. **101**(1), 83–89 (2004) 15221992 10.1002/cncr.20327

[40] A.K. Behera et al., Functional interplay between YY1 and CARM1 promotes oral carcinogenesis. Oncotarget. **10**(38), 3709–3724 (2019) 31217904 10.18632/oncotarget.26984 PMC6557205

[41] R. Elakoum et al., CARM1 and PRMT1 are dysregulated in lung cancer without hierarchical features. Biochimie. **97**, 210–218 (2014) 24211191 10.1016/j.biochi.2013.10.021

[42] K. Limm et al., Deregulation of protein methylation in melanoma. Eur. J. Cancer. **49**(6), 1305–1313 (2013) 23265702 10.1016/j.ejca.2012.11.026

[43] K.B. O’Brien et al., CARM1 is required for proper control of proliferation and differentiation of pulmonary epithelial cells. Development. **137**(13), 2147–2156 (2010) 20530543 10.1242/dev.037150 PMC2882134

[44] J. Wu et al., A role for CARM1-mediated histone H3 arginine methylation in protecting histone acetylation by releasing corepressors from chromatin. PLoS One. **7**(6), e34692 (2012) 22723830 10.1371/journal.pone.0034692 PMC3377634

[45] Y.H. Lee, M.T. Bedford, M.R. Stallcup, Regulated recruitment of tumor suppressor BRCA1 to the p21 gene by coactivator methylation. Genes Dev. **25**(2), 176–188 (2011) 21245169 10.1101/gad.1975811 PMC3022263

[46] S.B. Hu et al., Protein arginine methyltransferase CARM1 attenuates the paraspeckle-mediated nuclear retention of mRNAs containing IRAlus. Genes Dev. **29**(6), 630–645 (2015) 25792598 10.1101/gad.257048.114 PMC4378195

[47] S.L. Jacques et al., CARM1 preferentially methylates H3R17 over H3R26 through a Random Kinetic mechanism. Biochemistry. **55**(11), 1635–1644 (2016) 26848779 10.1021/acs.biochem.5b01071

[48] D. Cheng et al., Genetic evidence for partial redundancy between the arginine methyltransferases CARM1 and PRMT6. J. Biol. Chem. **295**(50), 17060–17070 (2020) 33008887 10.1074/jbc.RA120.014704 PMC7863876

[49] M. Nie et al., CARM1-mediated methylation of protein arginine methyltransferase 5 represses human γ-globin gene expression in erythroleukemia cells. J. Biol. Chem. **293**(45), 17454–17463 (2018) 30257864 10.1074/jbc.RA118.004028 PMC6231142

[50] J. Wu, W. Xu, Histone H3R17me2a mark recruits human RNA polymerase-associated factor 1 complex to activate transcription. Proc. Natl. Acad. Sci. U S A **109**(15), 5675–5680 (2012) 22451921 10.1073/pnas.1114905109 PMC3326481

[51] U.M. Bauer et al., Methylation at arginine 17 of histone H3 is linked to gene activation. EMBO Rep. **3**(1), 39–44 (2002) 11751582 10.1093/embo-reports/kvf013 PMC1083932

[52] G. Yang et al., Base-editing-mediated R17H substitution in histone H3 reveals methylation-dependent regulation of Yap Signaling and early mouse embryo development. Cell. Rep. **26**(2), 302–312e4 (2019) 30625312 10.1016/j.celrep.2018.12.046

[53] E. Zika et al., Interplay among coactivator-associated arginine methyltransferase 1, CBP, and CIITA in IFN-gamma-inducible MHC-II gene expression. Proc. Natl. Acad. Sci. U S A **102**(45), 16321–16326 (2005) 16254053 10.1073/pnas.0505045102 PMC1283426

[54] D. Chen et al., Regulation of transcription by a protein methyltransferase. Science. **284**(5423), 2174–2177 (1999) 10381882 10.1126/science.284.5423.2174

[55] P. Yi et al., Structural and functional impacts of ER Coactivator Sequential Recruitment. Mol. Cell. **67**(5), 733–743e4 (2017) 28844863 10.1016/j.molcel.2017.07.026 PMC5657569

[56] D.G. Ceschin et al., Methylation specifies distinct estrogen-induced binding site repertoires of CBP to chromatin. Genes Dev. **25**(11), 1132–1146 (2011) 21632823 10.1101/gad.619211 PMC3110952

[57] K.J. Veazey et al., CARM1 inhibition reduces histone acetyltransferase activity causing synthetic lethality in CREBBP/EP300-mutated lymphomas. Leukemia. **34**(12), 3269–3285 (2020) 32576962 10.1038/s41375-020-0908-8 PMC7688486

[58] Z. Liu et al., NFYC-37 promotes tumor growth by activating the mevalonate pathway in bladder cancer. Cell. Rep. **42**(8), 112963 (2023) 37561631 10.1016/j.celrep.2023.112963

[59] S. Frietze et al., CARM1 regulates estrogen-stimulated breast cancer growth through up-regulation of E2F1. Cancer Res. **68**(1), 301–306 (2008) 18172323 10.1158/0008-5472.CAN-07-1983

[60] L. Wang et al., CARM1 methylates chromatin remodeling factor BAF155 to enhance tumor progression and metastasis. Cancer Cell. **25**(1), 21–36 (2014) 24434208 10.1016/j.ccr.2013.12.007 PMC4004525

[61] W. An, J. Kim, R.G. Roeder, Ordered cooperative functions of PRMT1, p300, and CARM1 in transcriptional activation by p53. Cell. **117**(6), 735–748 (2004) 15186775 10.1016/j.cell.2004.05.009

[62] I.M. Fingerman, S.D. Briggs, *p53-mediated transcriptional activation: from test tube to cell* Cell, 2004. 117(6): pp. 690-1 10.1016/j.cell.2004.05.021 15186770

[63] K.Y. Kim et al., PRMT4-mediated arginine methylation negatively regulates retinoblastoma tumor suppressor protein and promotes E2F-1 dissociation. Mol. Cell. Biol. **35**(1), 238–248 (2015) 25348716 10.1128/MCB.00945-14 PMC4295381

[64] T. Fujiwara et al., CARM1 regulates proliferation of PC12 cells by methylating HuD. Mol. Cell. Biol. **26**(6), 2273–2285 (2006) 16508003 10.1128/MCB.26.6.2273-2285.2006 PMC1430293

[65] D. Cheng et al., The arginine methyltransferase CARM1 regulates the coupling of transcription and mRNA processing. Mol. Cell. **25**(1), 71–83 (2007) 17218272 10.1016/j.molcel.2006.11.019

[66] F. Haque, T. Honjo, N.A. Begum, XLID syndrome gene Med12 promotes ig isotype switching through chromatin modification and enhancer RNA regulation. Sci. Adv. **8**(47), eadd1466 (2022) 36427307 10.1126/sciadv.add1466 PMC9699684

[67] T. Abeywardana et al., CARM1 suppresses de novo serine synthesis by promoting PKM2 activity. J. Biol. Chem. **293**(39), 15290–15303 (2018) 30131339 10.1074/jbc.RA118.004512 PMC6166735

[68] F. Liu et al., PKM2 methylation by CARM1 activates aerobic glycolysis to promote tumorigenesis. Nat. Cell. Biol. **19**(11), 1358–1370 (2017) 29058718 10.1038/ncb3630 PMC5683091

[69] L. Zhang et al., Arginine methylation of PPP1CA by CARM1 regulates glucose metabolism and affects osteogenic differentiation and osteoclastic differentiation. Clin. Transl Med. **13**(9), e1369 (2023) 37649137 10.1002/ctm2.1369 PMC10468565

[70] S. Lombardi et al., Targeting fatty acid reprogramming suppresses CARM1-expressing Ovarian Cancer. Cancer Res. Commun. **3**(6), 1067–1077 (2023) 37377614 10.1158/2767-9764.CRC-23-0030 PMC10281290

[71] Y. Liu et al., A C9orf72-CARM1 axis regulates lipid metabolism under glucose starvation-induced nutrient stress. Genes Dev. **32**(21–22), 1380–1397 (2018) 30366907 10.1101/gad.315564.118 PMC6217731

[72] S.C. Wang et al., CARM1/PRMT4 is necessary for the glycogen gene expression programme in skeletal muscle cells. Biochem. J. **444**(2), 323–331 (2012) 22428544 10.1042/BJ20112033

[73] Y. Wang et al., PRMT4 promotes ferroptosis to aggravate doxorubicin-induced cardiomyopathy via inhibition of the Nrf2/GPX4 pathway. Cell. Death Differ. **29**(10), 1982–1995 (2022) 35383293 10.1038/s41418-022-00990-5 PMC9525272

[74] J. Guo et al., Arginine methylation of ribose-5-phosphate isomerase A senses glucose to promote human colorectal cancer cell survival. Sci. China Life Sci. **63**(9), 1394–1405 (2020) 32157557 10.1007/s11427-019-1562-y

[75] H.R. Shin et al., Epigenetic and transcriptional regulation of autophagy. Autophagy. **12**(11), 2248–2249 (2016) 27487449 10.1080/15548627.2016.1214780 PMC5103355

[76] Y. Liu, J. Wang, C9orf72-dependent lysosomal functions regulate epigenetic control of autophagy and lipid metabolism. Autophagy. **15**(5), 913–914 (2019) 30767689 10.1080/15548627.2019.1580106 PMC6526819

[77] Y.S. Yu et al., Pontin arginine methylation by CARM1 is crucial for epigenetic regulation of autophagy. Nat. Commun. **11**(1), 6297 (2020) 33293536 10.1038/s41467-020-20080-9 PMC7722926

[78] K. Zhou et al., TFE3, a potential therapeutic target for spinal cord Injury via augmenting autophagy flux and alleviating ER stress. Theranostics. **10**(20), 9280–9302 (2020) 32802192 10.7150/thno.46566 PMC7415792

[79] S. Yang et al., CARM1 promotes gastric cancer progression by regulating TFE3 mediated autophagy enhancement through the cytoplasmic AMPK-mTOR and nuclear AMPK-CARM1-TFE3 signaling pathways. Cancer Cell. Int. **22**(1), 102 (2022) 35246137 10.1186/s12935-022-02522-0 PMC8895580

[80] M. Covic et al., Arginine methyltransferase CARM1 is a promoter-specific regulator of NF-kappaB-dependent gene expression. Embo j. **24**(1), 85–96 (2005) 15616592 10.1038/sj.emboj.7600500 PMC544912

[81] S. Jayne, K.M. Rothgiesser, M.O. Hottiger, CARM1 but not its enzymatic activity is required for transcriptional coactivation of NF-kappaB-dependent gene expression. J. Mol. Biol. **394**(3), 485–495 (2009) 19769987 10.1016/j.jmb.2009.09.032

[82] L. Espinosa et al., The Notch/Hes1 pathway sustains NF-κB activation through CYLD repression in T cell leukemia. Cancer Cell. **18**(3), 268–281 (2010) 20832754 10.1016/j.ccr.2010.08.006 PMC2963042

[83] K. Hein et al., Site-specific methylation of Notch1 controls the amplitude and duration of the Notch1 response. Sci. Signal. **8**(369), ra30 (2015) 25805888 10.1126/scisignal.2005892

[84] Q. Chen et al., LYL1 facilitates AETFC assembly and gene activation by recruiting CARM1 in t(8;21) AML. Proc. Natl. Acad. Sci. U S A **119**(42), e2213718119 (2022) 36215477 10.1073/pnas.2213718119 PMC9586329

[85] S.M. Greenblatt et al., CARM1 is essential for myeloid leukemogenesis but dispensable for normal hematopoiesis. Cancer Cell. **33**(6), 1111–1127e5 (2018) 29894694 10.1016/j.ccell.2018.05.007 PMC6191185

[86] S. Majumder et al., Involvement of arginine methyltransferase CARM1 in androgen receptor function and prostate cancer cell viability. Prostate. **66**(12), 1292–1301 (2006) 16705743 10.1002/pros.20438

[87] G. Gao et al., The NFIB/CARM1 partnership is a driver in preclinical models of small cell lung cancer. Nat. Commun. **14**(1), 363 (2023) 36690626 10.1038/s41467-023-35864-y PMC9870865

[88] D.I. Kim et al., High-glucose-induced CARM1 expression regulates apoptosis of human retinal pigment epithelial cells via histone 3 arginine 17 dimethylation: role in diabetic retinopathy. Arch. Biochem. Biophys. **560**, 36–43 (2014) 25072916 10.1016/j.abb.2014.07.021

[89] E.J. Kim et al., BAF155 methylation drives metastasis by hijacking super-enhancers and subverting anti-tumor immunity. Nucleic Acids Res. **49**(21), 12211–12233 (2021) 34865122 10.1093/nar/gkab1122 PMC8643633

[90] J. Lee et al., ESRP1-regulated isoform switching of LRRFIP2 determines metastasis of gastric cancer. Nat. Commun. **13**(1), 6274 (2022) 36307405 10.1038/s41467-022-33786-9 PMC9616898

[91] C. Xu et al., CDCA4 suppresses epithelial-mesenchymal transtion (EMT) and metastasis in non-small cell lung cancer through modulating autophagy. Cancer Cell. Int. **21**(1), 48 (2021) 33436008 10.1186/s12935-021-01754-w PMC7802205

[92] X.C. Cai et al., A chemical probe of CARM1 alters epigenetic plasticity against breast cancer cell invasion. Elife, 2019. 8 10.7554/eLife.47110 PMC6917500 31657716

[93] L. Wang et al., MED12 methylation by CARM1 sensitizes human breast cancer cells to chemotherapy drugs. Sci. Adv. **1**(9), e1500463 (2015) 26601288 10.1126/sciadv.1500463 PMC4646802

[94] F. Shu et al., Epigenetic and post-translational modifications in autophagy: biological functions and therapeutic targets. Signal. Transduct. Target. Ther. **8**(1), 32 (2023) 36646695 10.1038/s41392-022-01300-8 PMC9842768

[95] M. Paquette et al., AMPK-dependent phosphorylation is required for transcriptional activation of TFEB and TFE3. Autophagy. **17**(12), 3957–3975 (2021) 33734022 10.1080/15548627.2021.1898748 PMC8726606

[96] S. Bertozzi et al., TFEB, SIRT1, CARM1, Beclin-1 expression and PITX2 methylation in breast cancer chemoresistance: a retrospective study. BMC Cancer. **21**(1), 1118 (2021) 34663249 10.1186/s12885-021-08844-y PMC8524961

[97] S. Kumar et al., CARM1 inhibition enables immunotherapy of resistant tumors by Dual Action on Tumor Cells and T cells. Cancer Discov. **11**(8), 2050–2071 (2021) 33707234 10.1158/2159-8290.CD-20-1144 PMC8338742

[98] A.E. Drew et al., Identification of a CARM1 inhibitor with potent in Vitro and in vivo activity in preclinical models of multiple myeloma. Sci. Rep. **7**(1), 17993 (2017) 29269946 10.1038/s41598-017-18446-z PMC5740082

[99] K. Nakayama et al., TP-064, a potent and selective small molecule inhibitor of PRMT4 for multiple myeloma. Oncotarget. **9**(26), 18480–18493 (2018) 29719619 10.18632/oncotarget.24883 PMC5915086

[100] Z. Zhang et al., Structure-based Discovery of Potent CARM1 inhibitors for solid Tumor and Cancer Immunology Therapy. J. Med. Chem. **64**(22), 16650–16674 (2021) 34781683 10.1021/acs.jmedchem.1c01308

[101] T. Ran et al., Virtual screening with a structure-based Pharmacophore Model to identify small-molecule inhibitors of CARM1. J. Chem. Inf. Model. **59**(1), 522–534 (2019) 30607947 10.1021/acs.jcim.8b00610

[102] Z. Guo et al., Design and synthesis of Potent, selective inhibitors of protein arginine methyltransferase 4 against Acute myeloid leukemia. J. Med. Chem. **62**(11), 5414–5433 (2019) 31117515 10.1021/acs.jmedchem.9b00297

[103] M. Békés, D.R. Langley, C.M. Crews, PROTAC targeted protein degraders: the past is prologue. Nat. Rev. Drug Discov. **21**(3), 181–200 (2022) 35042991 10.1038/s41573-021-00371-6 PMC8765495

[104] H. Xie et al., Development of Potent and Selective Coactivator-Associated Arginine methyltransferase 1 (CARM1) degraders. J. Med. Chem. **66**(18), 13028–13042 (2023) 37703322 10.1021/acs.jmedchem.3c00982 PMC10775954

[105] J.I. Bang et al., The effect of cell penetrating peptide-conjugated coactivator-associated arginine methyltransferase 1 (CPP-CARM1) on the cloned mouse embryonic development. Sci. Rep. **8**(1), 16721 (2018) 30425285 10.1038/s41598-018-35077-0 PMC6233168

[106] J. Jo et al., Regulation of differentiation potential of human mesenchymal stem cells by intracytoplasmic delivery of coactivator-associated arginine methyltransferase 1 protein using cell-penetrating peptide. Stem Cells. **30**(8), 1703–1713 (2012) 22696466 10.1002/stem.1146

[107] S. Karakashev et al., EZH2 inhibition sensitizes CARM1-High, homologous recombination proficient ovarian cancers to PARP inhibition. Cancer Cell. **37**(2), 157–167 (2020)..e6 32004442 10.1016/j.ccell.2019.12.015 PMC7155421

[108] C.S. Lim, D.L. Alkon, Protein kinase C stimulates HuD-mediated mRNA stability and protein expression of neurotrophic factors and enhances dendritic maturation of hippocampal neurons in culture. Hippocampus. **22**(12), 2303–2319 (2012) 22736542 10.1002/hipo.22048

[109] X. Li et al., Oxidative stress destabilizes protein arginine methyltransferase 4 via glycogen synthase kinase 3β to impede lung epithelial cell migration. Am. J. Physiol. Cell. Physiol. **313**(3), C285–c294 (2017) 28637674 10.1152/ajpcell.00073.2017 PMC5625095

[110] C. Li et al., Nuclear AMPK regulated CARM1 stabilization impacts autophagy in aged heart. Biochem. Biophys. Res. Commun. **486**(2), 398–405 (2017) 28315332 10.1016/j.bbrc.2017.03.053

[111] Y. Zhang et al., Structural studies provide New insights into the role of lysine acetylation on substrate recognition by CARM1 and inform the design of potent peptidomimetic inhibitors. Chembiochem. **22**(24), 3469–3476 (2021) 34569136 10.1002/cbic.202100506 PMC9293414

[112] T.A. Klink et al., Development and validation of a generic fluorescent methyltransferase activity assay based on the transcreener AMP/GMP assay. J. Biomol. Screen. **17**(1), 59–70 (2012) 21956169 10.1177/1087057111421624 PMC3707307

[113] H. Hu et al., Small molecule inhibitors of protein arginine methyltransferases. Expert Opin. Investig. Drugs. **25**(3), 335–358 (2016) 26789238 10.1517/13543784.2016.1144747 PMC4929062

[114] Y. Ohta et al., Ratiometric assay of CARM1 activity using a FRET-based fluorescent probe. Bioorg. Med. Chem. Lett. **29**(22), 126728 (2019) 31607607 10.1016/j.bmcl.2019.126728

[115] S. Suresh, S. Huard, T. Dubois, CARM1/PRMT4: making its Mark beyond its function as a transcriptional Coactivator. Trends Cell. Biol. **31**(5), 402–417 (2021) 33485722 10.1016/j.tcb.2020.12.010

[116] H. Naeem et al., The activity and stability of the transcriptional coactivator p/CIP/SRC-3 are regulated by CARM1-dependent methylation. Mol. Cell. Biol. **27**(1), 120–134 (2007) 17043108 10.1128/MCB.00815-06 PMC1800659

[117] Q. Feng et al., Signaling within a coactivator complex: methylation of SRC-3/AIB1 is a molecular switch for complex disassembly. Mol. Cell. Biol. **26**(21), 7846–7857 (2006) 16923966 10.1128/MCB.00568-06 PMC1636757

[118] W. Xu et al., A transcriptional switch mediated by cofactor methylation. Science. **294**(5551), 2507–2511 (2001) 11701890 10.1126/science.1065961

[119] M. Chevillard-Briet, D. Trouche, L. Vandel, Control of CBP co-activating activity by arginine methylation. Embo j. **21**(20), 5457–5466 (2002) 12374746 10.1093/emboj/cdf548 PMC129080

[120] B.T. Schurter et al., Methylation of histone H3 by coactivator-associated arginine methyltransferase 1. Biochemistry. **40**(19), 5747–5756 (2001) 11341840 10.1021/bi002631b

[121] Y. Yang et al., TDRD3 is an effector molecule for arginine-methylated histone marks. Mol. Cell. **40**(6), 1016–1023 (2010) 21172665 10.1016/j.molcel.2010.11.024 PMC3090733

[122] H. Ma et al., Hormone-dependent, CARM1-directed, arginine-specific methylation of histone H3 on a steroid-regulated promoter. Curr. Biol. **11**(24), 1981–1985 (2001) 11747826 10.1016/s0960-9822(01)00600-5

[123] M. Goolam et al., Heterogeneity in Oct4 and Sox2 targets biases cell fate in 4-Cell mouse embryos. Cell. **165**(1), 61–74 (2016) 27015307 10.1016/j.cell.2016.01.047 PMC4819611

[124] Z. Zhang et al., Crosstalk between histone modifications indicates that inhibition of arginine methyltransferase CARM1 activity reverses HIV latency. Nucleic Acids Res. **45**(16), 9348–9360 (2017) 28637181 10.1093/nar/gkx550 PMC5766202

[125] F. Casadio et al., H3R42me2a is a histone modification with positive transcriptional effects. Proc. Natl. Acad. Sci. U S A **110**(37), 14894–14899 (2013) 23980157 10.1073/pnas.1312925110 PMC3773778

[126] W.W. Gao et al., Arginine methylation of HSP70 regulates retinoid acid-mediated RARβ2 gene activation. Proc. Natl. Acad. Sci. U S A **112**(26), E3327–E3336 (2015) 26080448 10.1073/pnas.1509658112 PMC4491752

[127] J. Bao et al., The arginine methyltransferase CARM1 represses p300•ACT•CREMτ activity and is required for spermiogenesis. Nucleic Acids Res. **46**(9), 4327–4343 (2018) 29659998 10.1093/nar/gky240 PMC5961101

[128] Y.H. Lee et al., Regulation of coactivator complex assembly and function by protein arginine methylation and demethylimination. Proc. Natl. Acad. Sci. U S A **102**(10), 3611–3616 (2005) 15731352 10.1073/pnas.0407159102 PMC553305

[129] Y. Kawabe et al., Carm1 regulates Pax7 transcriptional activity through MLL1/2 recruitment during asymmetric satellite stem cell divisions. Cell. Stem Cell. **11**(3), 333–345 (2012) 22863532 10.1016/j.stem.2012.07.001 PMC3438319

[130] F. Wang et al., Nup54-induced CARM1 nuclear importation promotes gastric cancer cell proliferation and tumorigenesis through transcriptional activation and methylation of Notch2. Oncogene. **41**(2), 246–259 (2022) 34725461 10.1038/s41388-021-02078-9

[131] H.Y. Zhao et al., CARM1 mediates modulation of Sox2. PLoS One. **6**(10), e27026 (2011) 22046437 10.1371/journal.pone.0027026 PMC3203945

[132] J. Lee, M.T. Bedford, PABP1 identified as an arginine methyltransferase substrate using high-density protein arrays. EMBO Rep. **3**(3), 268–273 (2002) 11850402 10.1093/embo-reports/kvf052 PMC1084016

[133] D. Cheng, M.T. Bedford, Xenoestrogens regulate the activity of arginine methyltransferases. Chembiochem. **12**(2), 323–329 (2011) 21243720 10.1002/cbic.201000522 PMC3142315

[134] R.J. 3 Sims et al., The C-terminal domain of RNA polymerase II is modified by site-specific methylation. Science. **332**(6025), 99–103 (2011) 10.1126/science.1202663 PMC3773223 21454787

[135] H. Li et al., Lipopolysaccharide-induced methylation of HuR, an mRNA-stabilizing protein, by CARM1. Coactivator-associated arginine methyltransferase. J. Biol. Chem. **277**(47), 44623–44630 (2002) 12237300 10.1074/jbc.M206187200

[136] S.F. Battaglia-Hsu et al., Inherited disorders of cobalamin metabolism disrupt nucleocytoplasmic transport of mRNA through impaired methylation/phosphorylation of ELAVL1/HuR. Nucleic Acids Res. **46**(15), 7844–7857 (2018) 30016500 10.1093/nar/gky634 PMC6125644

[137] V. Calvanese et al., Sirtuin 1 regulation of developmental genes during differentiation of stem cells. Proc. Natl. Acad. Sci. U S A **107**(31), 13736–13741 (2010) 20631301 10.1073/pnas.1001399107 PMC2922228

[138] J. Kim et al., Loss of CARM1 results in hypomethylation of thymocyte cyclic AMP-regulated phosphoprotein and deregulated early T cell development. J. Biol. Chem. **279**(24), 25339–25344 (2004) 15096520 10.1074/jbc.M402544200

